# DeFiNe: an optimisation-based method for robust disentangling of filamentous networks

**DOI:** 10.1038/srep18267

**Published:** 2015-12-15

**Authors:** David Breuer, Zoran Nikoloski

**Affiliations:** 1 Systems Biology and Mathematical Modeling, Max Planck Institute of Molecular Plant Physiology, Am Muehlenberg 1, 14476 Potsdam, Germany

## Abstract

Thread-like structures are pervasive across scales, from polymeric proteins to root systems to galaxy filaments, and their characteristics can be readily investigated in the network formalism. Yet, network links usually represent only parts of filaments, which, when neglected, may lead to erroneous conclusions from network-based analyses. The existing alternatives to detect filaments in network representations require tuning of parameters over a large range of values and treat all filaments equally, thus, precluding automated analysis of diverse filamentous systems. Here, we propose a fully automated and robust optimisation-based approach to detect filaments of consistent intensities and angles in a given network. We test and demonstrate the accuracy of our solution with contrived, biological, and cosmic filamentous structures. In particular, we show that the proposed approach provides powerful automated means to study properties of individual actin filaments in their network context. Our solution is made publicly available as an open-source tool, “DeFiNe”, facilitating decomposition of any given network into individual filaments.

Many network-like structures in nature are composed of filaments forming intricate interconnected arrays across different scales of organisation. For instance, filamentous structures can be observed in networks of cellulose polymers in the primary cell wall of plants and algae^1^,^2^, cytoskeletal networks of actin filaments or microtubules in cells across all domains of life^3^,^4^,^5^, networks of neurons^6^,^7^, root systems^8^,^9^,^10^, as well as solar prominences^11^,^12^ and galaxy clusters^13^,^14^,^15^,^16^. Network-based studies of these structures have already elucidated important aspects such as the mechanics of cellulose networks^1^,^17^, transport on cytoskeletal actin networks^18^,^19^, and connectivity patterns in the brain^7^,^20^,^21^. However, the network links usually correspond to segments of the filaments; therefore, the classical network-based analysis neglects the identities of individual filaments. A few powerful exceptions have recently started to emerge^22^,^23^ which may identify multiple segments that belong to the same filament; yet, since these studies do not capture filament overlaps, filaments are still broken into potentially multiple fragments. Characterisation of the mechanical-24,25,26, transport-19,27, and information-transmission related properties^28^,^29^ in such network representations may hence lead to erroneous conclusions due to their differences within and between filaments. Thus, analysis of filamentous structures rests upon accurate identification of individual filaments.

Since most of the filamentous structures in natural and man-made systems are studied by using imaging technologies, filaments are identified either directly from the imaging data or from networks extracted from these data (see Table 1 for succinct review). In the first class of approaches, a texture-based method is employed to infer the overall orientation of objects in an image section^30^. However, this method cannot be employed to pinpoint individual filaments. Another method decomposes entire images of filamentous structures into linear segments based on a linear programming formulation^31^. While this method utilises few parameters (e.g., number of filaments), it only models and extracts a representative set of linear filaments. Moreover, filaments have been modelled as linear segments, detected by co-localisation with a parallel grid at different orientations and by using manually chosen intensity thresholds along a filament^32^. While this method is fast and useful for extracting linear filaments (e.g., microtubules), it does not capture bent or tangled filaments and necessitates manual parameter selection. Alternatively, tracing- and tracking-based methods which start from one or multiple image points and predict neighbouring points on a putative filament through optimisation of an energy function are powerful methods for filament identification. Although these algorithms have led to great insights, especially into the connectome, they typically require user input and do not capture overlapping filaments^33^,^34^,^35^,^36^. Using a similar approach, open contour-based methods employ deformable curve models that elongate and align according to an energy functional to match the target filaments. Recent advances in open contour-based approaches enable fully automated filament detection^22^,^23^, but can account for the overlap of only few filaments at the expense of parameter tuning^37^.

The second class of approaches for disentangling filamentous structures employs a two-step procedure: First, weighted networks are extracted from image data from different systems and imaging sources. There is a large variety of algorithms for this task^33^,^38^,^39^,^40^ which vary, in particular, in the number of parameters. Some of the methods from the first class, presented above, may also be used to obtain such network representations (e.g.^23^,^36^). Second, the given, weighted networks are decomposed into filaments. The two existing methods for this task^38^,^41^ define specific junctions for bifurcations and crossings of filaments, depending on the distances between nodes, and assign filament identities according to manually chosen angle thresholds between incoming and outgoing edges. In particular, they strongly restrict the potential overlap of filaments and, due to the angle constraints, allow only crossing but no touching filaments. Most importantly, these methods require manual parameter selection and do not take into account filament intensity/thickness. We note that the step of decomposing a given network may also be beneficially applied to networks obtained, e.g., by open contour-based approaches in which filaments have been fragmented due to omission of filament overlaps^22^,^23^.

Here, we propose a robust approach to decompose a weighted network into an optimal set of individual filaments. Therefore, our approach addresses the second step in the second class of approaches, presented above. The decomposition is based on a computationally difficult problem, referred to as filament cover problem (FCP), for which we propose suitable approximation algorithms. We test and demonstrate the accuracy of the findings from the approximation algorithms on artificial as well as biological and cosmic filamentous networks by comparison to manually obtained filament covers. In addition, we demonstrate that the proposed, fully automated solution allows facile characterisation of well-studied properties of individual filaments, for which alternative approaches require parameter tuning or time-consuming manual tracing. The proposed approach is implemented in a publicly available open-source tool, “DeFiNe” (**De**composing **Fi**lamentous **Ne**tworks), which can be used to decompose any given weighted network into a set of individual filaments for further analyses (http://mathbiol.mpimp-golm.mpg.de/DeFiNe/).

## Methods

In this section we introduce the mathematical formulation of our optimisation-based approach to decompose filamentous networks, demonstrate its computational intractability, and formulate a suitable approximation scheme. Moreover, we introduce new quality measures which take into account the underlying network structures for the comparison of the obtained filament decompositions with manual assignments used as a gold standard. Finally, we provide a brief overview of the studied data from different biological and physical systems. While we believe that these more technical explanations may promote a deeper understanding of our and related approaches, we encourage readers familiar with the aforementioned topics to proceed directly to the Results.

### Mathematical formulation of the filament cover problem

Any filamentous structure may be represented as a weighted geometric graph 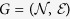 with 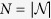 nodes and 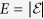 undirected, weighted edges. Edges represent filament segments and their intensities or thicknesses are reflected by their weights *w*_*e*_, 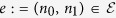 and 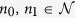. Nodes represent endpoints of filament segments and their positions are denoted by *v*_*n*_, 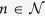, whereby, typically, 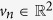 or 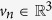 for networks extracted from image data.

We naturally represent a filament by an edge-path, *p* = (*e*_*p*,1_, …, *e*_*p*,*P*_), 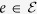, i.e., by an ordered sequence of *P* = |*p*| adjacent edges, where *e*_*p*,*i*_ denotes the *i*-th edge of filament *p*. The quality of a given filament *p* is assessed by the pairwise filament roughness


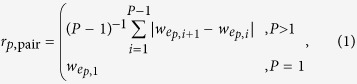


where 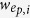 denotes the weight of the *i*-th edge in filament *p*. The pairwise filament roughness is small if the edge weights along a filament vary smoothly, as expected for natural filaments (but cf. Discussion). For filaments that consist of one edge only, their roughness is given by their edge weight to increase the flexibility of our approach (cf. Supplemental Material S1). Other roughness measures may be readily introduced that take into account filament thicknesses or alignments. As an additional example, we study the all-to-all filament roughness


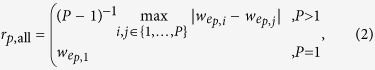


which is the average maximal difference between any two edge weights in a filament *p*, and again the original weight of the edge is used for a filament of length one. Taking into account that most filaments are only moderately bent, we further consider the maximal filament deflection angle between adjacent edges of a path *p*,


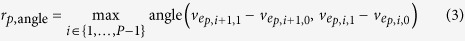


where 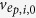 and 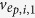 denote the positions of the start and end nodes of the *i*-th edge of filament *p*, respectively. Moreover, 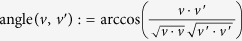 is the Euclidean angle of two vectors *v* and *v*′ and *r*_*p*,angle_ = 0° corresponds to perfectly straight alignment.

The optimal decomposition of a network into individual, smooth filaments then corresponds to solving our filament cover problem (FCP; cf. Supplemental Material S1 for an overview of related cover problems):

Given a graph 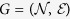 and the set 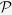 of all edge-paths in *G* with roughnesses *r*_*p*_, 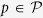:

Find a subset 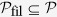 with minimal total (or average) roughness *R* such that each element in 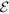 is covered (at least) once.

Here, edges that are covered by more than one path naturally correspond to filament overlaps. Minimising the average instead of the total roughness yields shorter filaments, as appropriate for some networks (cf. Supplemental Material S1).

### Computational intractability of the filament cover problem and approximation algorithm

The FCP is computationally intractable on general and even planar graphs (cf. Supplemental Material S2 for motivation and proof). Graphs generated from two-dimensional image data are planar by construction^39^,^40^. The proof is by reduction from the well-studied Hamiltonian path problem which asks, for a given network, whether there is a sequence of adjacent nodes that includes each node exactly once, and which is known to be intractable on planar graphs^42^. Moreover, we outline an algorithm for solving the FCP in polynomial time on trees (cf. Supplemental Material S3).

Since the FCP is computationally intractable on general and even planar graphs, we devise an approximation scheme by formulating the FCP as a fractional integer linear program (cf. Supplemental Material S4 for motivation and details). For a given set 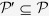 of input paths with pairwise filament roughnesses *r*_*p*_, 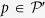, we solve:


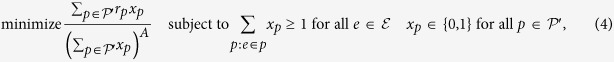


where we use *r*_*p*_ ∈ {*r*_*p*,pair_, *r*_*p*,all_} (Eqs. 1 or 2; referred to as *pair* and *all*). In the first line, *A* ∈ {0, 1} determines whether the total or the average roughness is minimised (*total*/*avg*). The inequality in the second line allows overlapping filaments and equality holds for an exact cover (*over*/*exact*). For *A* = 0, Eq. 4 is a binary linear program and for *A* = 1, the fractional problem Eq. 4 may be rewritten as a binary linear program (cf. Supplemental Material S4). Binary linear programs may be solved using well-established and efficient algorithms^43^,^44^.

To solve the FCP for a given network, we further need to collect a set of input paths 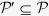. Since for a general graph it is not feasible to collect all paths 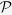 (cf. Supplemental Material S2), we propose two approaches (referred to as *RMST* and *BFS*): (1) We create *T* = 100 random minimal spanning trees (RMST) of *G* whose *N*(*N* − 1)/2 non-trivial, undirected paths are added to our set 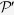. To obtain a RMST, each edge is assigned a randomly and uniformly distributed weight and the minimum spanning tree with respect to these weights is computed. (2) We perform a modified breadth-first search (BFS) on the nodes, stop the search for a path *p* when it violates the straightness criterion *r*_*p*,angle_ < 60° (cf. Eq. 3), and add all permitted paths to 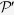. We note that for real-world filamentous graphs, the number of nodes and their degrees are constrained by the filament thickness, while the number of considered loops is further restricted by the straightness criterion, so that our heuristically modified BFS yields a representative set 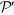 of paths in reasonable time. Moreover, we note that the 60°-criterion is introduced for computational reasons and provides a tolerant estimate for maximal bending of the studied real-world filaments which are typically less bent.

### Quality assessment of filament covers via structure-aware partition similarity measures

**Figure 1:**
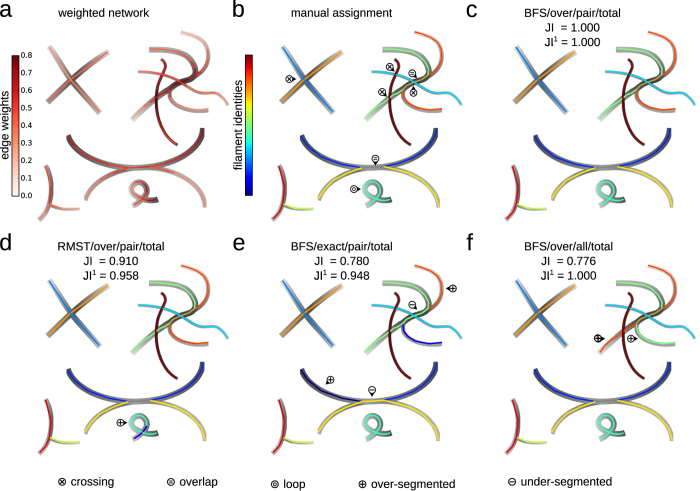
Filament covers of artificial network with crossings, overlaps, and a loop. (**a**) Weighted, artificial network extracted from the underlying drawing, with colour-coded edge weights representing the local image intensity. (**b**) Manual decomposition of the network into filaments with colour-coded indices. The filaments display crossings (⊗), overlaps (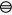), and a loop (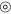). (**c**) Filament cover obtained by solving the FCP using the set of input paths generated by a modified breadth-first-search (*BFS*), allowing overlapping filaments (*over*), employing the pairwise roughness measure (*pair*), and by minimising the total roughness of the cover (*total*). The automatically obtained filament cover correctly captures crossings, overlaps, and loops, and agrees excellently with the manual assignment (similarity of the two filament covers is measured by the global Jaccard index, JI, and our modified, structure-aware Jaccard index, JI^1^, which reflect the fraction of pairs of all or only adjacent edges that are assigned to the same filament, respectively; here JI = JI^1^ = 1). The filament identities and colours are matched by solving an assignment problem whereby the total number of edges shared by two filaments, from the manual and automated partitioning, is maximised; the same assignment procedure is used for the remaining panels. (**d**) When using paths obtained from sampling random minimum spanning trees (*RMST*) for the FCP, the closed filament loop is not correctly detected and is over-segmented (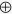). (**e**) When solving the exact FCP (*exact*), the loop is correctly detected. However, overlaps are neglected so that no two filaments share an edge, leading to over- and under-segmentation (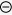). (**f**) When minimising the all-to-all filament roughness (*all*), two half-filaments are interchanged because the maximum weight difference is smaller along the altered filaments.

The accuracy of the filaments covers obtained by solving the FCP is assessed by comparison to manual filament assignments (cf. Fig. 1b). We quantify the similarity of the two partitions of the set of edges into (potentially overlapping) filaments using the variation of information, VI, the Jaccard index, JI, and the Rand index, RI,


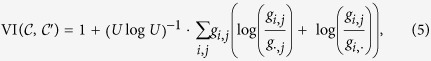



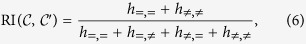



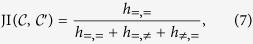


where 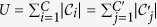, 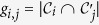, 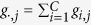, and 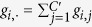
45,46,47. The contingency tables *h*_×,×′_, ×, ×′ ∈ {=, ≠}, provide the numbers of edge pairs which are in the same or different sets in the two partitions, respectively. While these classical measures are widely used^46^,^48^, they may generally yield opposing results and VI is not well-defined for overlapping partitions (cf. Supplemental Material S6). More severely, these measures do not take into account the structure of the graph underlying the partitions. To remedy this shortcoming, we introduce a suite of measures, the structure-aware Rand and Jaccard indices (cf. Eqs. 6 and 7),


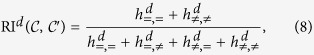



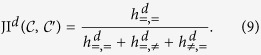


Here 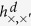, ×, × ′ ∈ {=, ≠}, 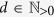, count the number of edge pairs which are in the same or different sets in the two partitions and which are separated by at most *d* nodes in *G* (cf. Supplemental Material S6 for details). Thus, RI^1^ and JI^1^ yield structure-aware measures of partition similarity that consider only the partition memberships of adjacent edges (local perspective), while RI^∞^ ≡ RI and JI^∞^ ≡ JI do no not take into account the positions of edges in the graph and reproduce the original measures (global perspective; cf. Supplemental Material S6 for an extensive comparison of similarity measures and intermediates between local and global perspective).

### Extraction of weighted networks from image data

We test our method to disentangle filamentous networks on various weighted, geometric networks extracted from image data. The network extraction procedure is similar to those proposed in^39^,^40^ (cf. Supplemental Material S5 for details). We analyse (1) two artificial networks extracted from drawn filamentous patterns, (2) two cytoskeletal networks from confocal microscope images of *Arabidopsis thaliana* hypocotyl actin cytoskeletons^49^, (3) 100 additional cytoskeletal networks from a movie over 200s from the same experimental setup (4), two neural networks from a fluorescence microscopy image of a branching rat hippocampal neuron *in vitro*^50^ and a schematic of a cat retinal ganglion cell^51^, respectively, and (5) two cosmic networks obtained from images of simulated galaxy clusters^14^ (see Table 2 for an overview).

## Results

### Decomposing filamentous networks is a hard optimisation problem

A filamentous network is naturally represented as a weighted graph, whereby the links (i.e., edges) denote segments of filaments and the nodes represent the ends of the segments. The edge weights typically capture the intensity or thickness of the filament segments. In this network representation, a filament corresponds to a path given by an ordered sequence of adjacent edges. To identify individual filaments, we seek a decomposition of the set of edges into paths so that each edge is covered (i.e., belongs to at least one path). Edges belonging to more than one path naturally model filament overlaps. We will refer to such a decomposition as a filament cover. Since a filament cover is non-unique, we introduce a quality measure, called roughness, to assess the quality of each path and the cover itself. Here we mainly consider the pairwise filament roughness given by the average absolute value of weight differences between adjacent edges. This roughness measure quantifies how strongly the thickness varies along a filament and is typically small for biological filaments. Disentangling the filamentous network amounts to solving the filament cover problem (FCP): Find a set of paths of minimum sum of roughness values that covers the network (cf. Methods and Supplemental Material S1 for the mathematical formulation). The FCP formulation is quite versatile: For instance, instead of minimising the total roughness of the filament cover, we may minimise the average roughness. This optimisation objective favours shorter filaments and may be more appropriate for specific types of networks. Other roughness measures (e.g., considering the spatial alignment of edges to penalise filaments with strong curvature) are readily introduced and can be considered in a multi-objective optimisation approach (cf. Methods and Supplemental Material S1 for different measures).

While providing a well-defined approach towards disentangling filamentous networks, solving the FCP is computationally prohibitive. Indeed, we show that the FCP is intractable even on planar graphs (cf. Methods and Supplemental Material S2) which are used to represent filamentous structures extracted from 2D image data^39^,^40^. While the FCP is solvable in polynomial time on trees (cf. Supplemental Material S3), most biological filamentous structures are not tree-like as they contain loops^40^,^49^,^52^. Therefore, we propose suitable approximation schemes to the FCP for the considered networks (cf. Methods and Supplemental Material S4 for details and the mathematical formulation). The approximation schemes rely on collecting a large sample of paths in a given graph, followed by the computation of the roughness of each path. The paths are collected by performing a modified breadth-first search (BFS) or by sampling from random minimum spanning trees (RMST). Next, we write the FCP as classical set cover problem^53^ which aims at covering the set of edges with a subset of the collected paths of minimum total or average roughness. The set cover approximation of FCP can be formulated and solved as a (fractional) binary linear program for which well-established algorithms exist^43^. The output of the program is a set of paths which correspond to the individual filaments of the studied network. Summarising, the FCP may be solved with different options: The initial set of paths is obtained from a modified BFS (denoted by *BFS*) or sampling of RMSTs (*RMST*), the filaments may overlap (*over*) or not (*exact*), a pairwise (*pair*) or all-to-all filament roughness measure (*all*) is used, and the total (*total*) or average (*avg*) roughness is minimised. Since all these options are categorical, all possible 2^4^ = 16 combinations may be readily checked and no data-specific and computationally demanding gauging of continuous parameters is necessary, as is the case for related approaches^38^,^41^. We provide an open-source implementation of our approach, termed “DeFiNe” (**De**composing **Fi**lamentous **Ne**tworks), with a simple and user-friendly graphical user interface available at http://mathbiol.mpimp-golm.mpg.de/DeFiNe/. DeFiNe takes as input a weighted graph in the standard .gml file format^54^ and outputs a .gml graph with filament identities stored as edge colours as well as a standard, human-readable .csv-table of various individual filament measures for custom analyses.

### Disentangling artificial filamentous structures

To test the accuracy of our approach, we investigate an artificial network (Fig. 1a) of pre-specified filamentous structure (Fig. 1b; cf. Methods and Supplemental Material S4 for the extraction of the network; cf. Supplemental Material S9 for an overview of the different stages of our approach, from an images to a network to filaments). The network contains crossings and overlapping filaments as well as a loop (Fig. 1b, ⊗, 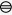, and 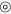 , respectively). First, we automatically decompose the weighted filamentous network by solving the FCP for a set of input paths from a modified BFS, allowing for overlaps, using the pairwise roughness measure, and minimising the total roughness of the cover (Fig. 1c, cf. Eq. 4). The filament identities and colours are matched by solving an assignment problem (cf.^55^,^56^) such that the total number of edges shared by two filaments, from the manual assignment and the automated cover, is maximised. The agreement between the automated cover and the manual assignment may be measured by classical partition similarity measures such as the Jaccard index JI which counts the fraction of edge pairs which are part of same filament^46^,^47^. However, JI does not take into account the structure of the underlying network. Hence, we introduced a new similarity measure, JI^1^, that considers only pairs of adjacent edges in each filament and thus incorporates the network structure (cf. Methods and and Supplemental Material S6 for details, a generalisation to JI^*d*^ that considers only pairs of edges which are separated by at most *d* nodes, and a comparison of various similarity measures). For our artificial network, solving the above FCP yields a decomposition which agrees excellently with the manual assignment (JI = JI^1^ = 1) as all filaments are correctly detected. Second, we choose a different set of input paths obtained from sampling RMSTs for solving th FCP (Fig. 1d). While most filaments are correctly detected, the loop (cf. Fig. 1b) is over-segmented (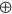) because it is not contained in the set of input paths in its entirety (due to looplessness of trees). Third, we solve the exact FCP which does not allow overlapping filaments (Fig. 1e). Expectedly, the agreement with the manual assignments is lower because filaments are over-segmented into disjoint segments and the supposedly overlapping parts are under-segmented (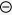), i.e., the respective edges are assigned to a single filament instead of multiple filaments. Finally, we employ the all-to-all roughness measure to assess the quality of the filaments (Fig. 1f, cf. Eq. 2). Filament crossings, overlaps, and the loop are again correctly detected but parts of two filaments are interchanged (cf. 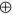). This is due to the intensity/thickness of the underlying filaments which is consistently higher for the new detected filaments which are therefore favoured by the all-to-all roughness measure. These test cases demonstrate the versatility and the accuracy of the proposed approach to decompose a given network into filaments.

In the analysis of many real-world filamentous structures, the knowledge of the underlying network structure is incomplete or the image data impede filament detection due to low signal-to-noise ratios. To investigate the effect of these obstacles on robust filament detection, we study two scenarios (Supplemental Material S7): In the first scenario, we remove a single edge from the network, recompute the optimal filament cover, and calculate its agreement with the manual filament assignment as measured by the structure-aware Jaccard index JI^1^. We repeat the procedure for all *E* edges and then proceed with the removal of *E* randomly chosen doubles of edges, triplets, up to subsets of 50 edges. As expected, the accuracy of the filament cover typically decreases with the number of removed edges, although removal of some specific edges even leads to an increase in accuracy. However, JI^1^ decreases very moderately by less than 0.001 per removed edge on average (cf. Supplemental Material S7). In the second scenario, we assess the robustness of our filament detection approach against noise by adding centred Gaussian noise of increasing standard deviation to the edge weights of the original network. For a given standard deviation, we obtain the optimal filament covers for 100 noisy network instances and compute their similarity, JI^1^, to the manual assignment. Again, as expected, the accuracy of the filament cover decreases with increasing noise, but only slightly. On average, increasing the noise by 1% of the original edge weights only decreases JI^1^ by less than 0.001. Moreover, we note that with increasing edge noise the accuracy of the filament cover approaches a constant, non-zero JI^1^ which reflects that some information about the filament structure maybe obtained from the topology of the network alone, irrespective of the edge weights (cf. Supplemental Material S7).

### Disentangling biological and cosmic filamentous structures

**Figure 2:**
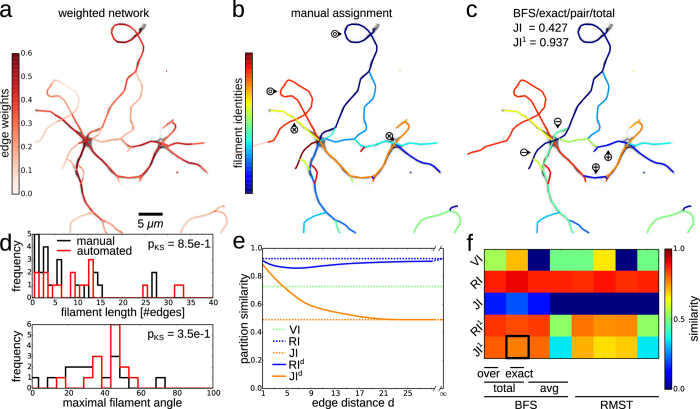
Filament covers and analyses of neuronal network. The weighted hippocampal neuronal network is automatically decomposed into filaments by solving the exact FCP (*exact*) for paths from a modified breadth-first search (*BFS*) and by minimising the total (*total*) pairwise filament roughness (*pair*). (**a**) Overlay of fluorescence microscopy image of hippocampal neurons and extracted network with colour-coded edge weights. (**b**) Manual decomposition of the neuronal network into filaments with colour-coded indices and crossings (⊗) and loops (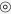). (**c**) Automated partitioning of the network obtained by solving the FCP displays good agreement with the manually obtained partitioning (JI^1^ close to 1, see panel (**e**) for details) with marked illustrative sites of over- (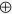) and under-segmentation (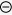). (**d**) Distributions of numbers of edges per filament (upper panel) as well as distributions of maximum filament angles (lower panel) are similar for manual (black) and automated decomposition (red; Kolmogorov-Smirnov test *p*_KS_ ≥ 0.05). (**e**) Different measures of similarity of manual and automated decompositions. The variation of information VI (dashed green) indicates moderate similarity but is not well-defined for general, overlapping decompositions. While the classical Jaccard index JI (dashed yellow) is of small value, the proposed structure-aware extension JI^*d*^ increases with decreasing *d*, i.e., when only edges are considered that are separated by at most *d* nodes (solid yellow). Moreover, while the classical Rand index RI (dashed blue) is of large value, the proposed structure-aware extension RI^*d*^ displays a non-monotonic dependence on *d* (solid blue). (**f**) Heat map of partition similarities for different similarity measures and options of the FCP, cf. Fig. 1 for a demonstration of the different options. The FCP options which yield the partition shown in (**c**) are marked by a black rectangle.

Since we demonstrated the power of the FCP-based approach on contrived filamentous structures, we next proceed with investigating real biological and cosmic filamentous structures (cf. Methods and Supplemental Material S5 for the extraction of the networks; cf. Supplemental Material S9 for an overview of the different stages of our approach). As a first illustrative example of a biological filamentous structure, we extract a weighted network from an image of a hippocampal neuron (Fig. 2a) and manually obtain a filament assignment with several crossings and loops (Fig. 2b, ⊗ and 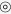, respectively). Solving the FCP (same options as in Fig. 1e) yields an automated decomposition which captures well the manual assignment, in particular the two loops (Fig. 2c, JI^1^ = 0.937). This is further supported by the distributions of filament lengths (measured by the numbers of edges) as well as the distributions of maximal filament angles (measured between adjacent edges), which are statistically indistinguishable between the manual assignment and the automated decomposition (Fig. 2d, black and red; Kolmogorov-Smirnov test *p*-value *p*_KS_ ≥ 0.05). A detailed analysis of the similarity of manual and automated decompositions shows that the classical Rand index RI^57^ overestimates the similarity, while the variation of information VI^58^ and the Jaccard index JI severely underestimate the similarity between the manual and automated decomposition when compared to the values of the here-proposed RI^1^ and JI^1^(Fig. 2e, dotted blue, green, and yellow). The latter measures take into consideration the network structure when comparing two network decompositions (Fig. 2e, solid blue and yellow). We would like to emphasise that the disparities in the estimations of RI and JI result from the consideration of distant, non-adjacent edges which are excluded in RI^1^ and JI^1^. In addition, we observe that RI^*d*^ and JI^*d*^ show a non-trivial dependence on the distance, *d*, between the considered edges, and coincide with the classical similarity measure for large enough distances, i.e., RI^∞^ ≡ RI and JI^∞^ ≡ JI (cf. Supplemental Material S6 for a detailed discussion).

Finally, different flavours of the FCP may be solved, as mentioned above, to obtain decompositions of varying similarity in comparison to the manual assignment (Fig. 2f). Solving the FCP with paths from the modified BFS, instead of RMSTs, yields consistently higher RI^1^- and JI^1^-values for the agreement with the manual assignment. This is due to the higher flexibility with respect to the treatment of loops. For the studied networks, a decomposition based on the minimisation of the total roughness yields higher RI^1^- and JI^1^-values in comparison to the minimisation of the average roughness. In addition, in terms of RI^1^ and JI^1^, covers allowing for overlaps yield better agreement with the manual assignment, in comparison to those in which each edge is covered by a single path. However, these expected trends are absent or even reversed for the classical similarity measures VI, RI, and JI (cf. Supplemental Material S6), which further justifies the usage of the here-proposed RI^1^ and JI^1^ for comparing decompositions of networks arising in other network-based analyses (cf. e.g.^59^).

**Figure 3:**
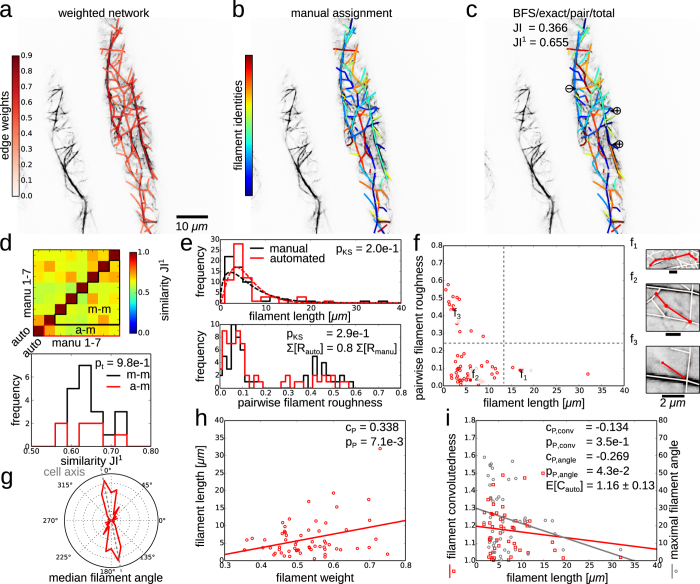
Filament covers and analyses of cytoskeletal network. The weighted cytoskeletal network is decomposed automatically by solving the exact FCP (*exact*) for paths from a modified breadth-first search (*BFS*) and by minimising the total (*total*) pairwise filament roughness (*pair*). (**a**) Overlay of confocal microscopy image of an actin cytoskeleton and extracted network with colour-coded edge weights. (**b**) Manual decomposition of the actin cytoskeleton into filaments with colour-coded indices. (**c**) The automated decomposition according to the FCP correctly assigns many of the filaments (JI^1^ = 0.655). Some occurrences of over- (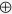) and under-segmentation (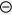) are marked. (**d**) Heat map of similarity between automated (cf. (**c**)) and seven manual decompositions (cf. e.g. (**b**); upper panel). The similarities between automated and manual decompositions (red, denoted by a-m) do not differ from similarities among the different manual decompositions (black, m-m; lower panel; cf. independent two-sample Student’s *t*-test *p*-value *p*_*t*_ ≥ 0.05). (**e**) Distribution of filament lengths for the manual (black) and automated solution (red) are similar (upper panel; cf. Kolmogorov-Smirnov test *p*-value *p*_KS_ ≥ 0.05). Maximum likelihood fits of gamma functions are shown as dashed lines. The distributions of pairwise filament roughnesses are similar (lower panel; cf. *p*-value *p*_KS_ ≥ 0.05), while the total roughness is smaller (cf. summed *R*-values) for the automated decomposition since it is minimised by the FCP. (**f**) Scatter plot of pairwise filament roughness versus filament length displays three regions, with representative examples f_1_ − f_3_ (solid dots): (f_1_) For long filaments (≥15μm), the roughness is moderate (<0.2), as expected for actin bundles; (f_2_) The majority of filaments is short (<15μm) and of moderate roughness; (f_3_) Some typically short filaments show a high roughness (≥ 0.2), namely those which are composed of one network edge only so that their roughness is given by the edge weight itself (cf. Eq. 1). (**g**) The distribution of median filament angles shows that the majority of filaments is aligned parallel to the cell axis (grey dashed line). (**h**) The filament length correlates with the filament weight (cf. linear regression and Pearson correlation coefficient *c*_*P*_ > 0 and *p*-value *p*_P_ < 0.05) (**i**) Scatter plot of filament convolutedness versus filament length shows a negative but non-significant correlation (cf. red squares, *c*_*P*,conv_ < 0, and *p*_P,conv_ ≥ 0.05) with an average convolutedness of E[*C*] = 1.16 ± 0.13. The maximum filament angle correlates negatively and significantly with the filament length (cf. grey circles, *c*_*P*,angle_ < 0, and *p*_P,angle_ < 0.05), indicating that longer (and thicker, cf. (**g**)) filaments are less curved.

As a second biological example, we investigate the filamentous structure of a plant actin cytoskeleton (Fig. 3a). We create seven manual assignments (one of which is shown in Fig. 3b) for a quantitative comparison with the automated decomposition (Fig. 3c, JI^1^ = 0.655; same options of the FCP as in Fig. 1e). The agreement of the automated decomposition with the manual assignment is good, despite several over- or under-segmented filaments (Fig. 3c, cf. 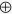 and 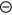). For a comprehensive assessment of this agreement, we compute the pairwise similarities between the automated and all seven manual filament decompositions (Fig. 3d, upper panel). By comparing the similarities between automated and manual decompositions to the similarities among the different manual decompositions, we find reassuringly that our automated solution is as good as any manual decomposition (Fig. 3d, lower panel, red and black, respectively; cf. independent two-sample Student’s *t*-test *p*-value *p*_*t*_ ≥ 0.05). The agreement between the automated decomposition and the reference manual assignment (cf. Fig. 3b) is further confirmed by statistical tests which demonstrate that the two distributions of filament lengths from manual assignment and automated decomposition do not statistically differ (Fig. 3e, upper panel, black and red histograms; cf. *p*_KS_ ≥ 0.05). In addition, our results indicate that the filament lengths may be described by a gamma distribution (Fig. 3e, upper panel, dashed lines; maximal likelihood fits of normal, Weibull, and Rayleigh distributions yield higher values for the Akaike information criterion^60^), in agreement with theoretical and experimental studies^61^,^62^. Moreover, the distributions of average pairwise filament roughnesses do not differ between manual assignment and automated decomposition (Fig. 3e, lower panel; cf. *p*_KS_ ≥ 0.05). We note that the sum of filament roughnesses, *R*, is larger for the manual assignment of filaments than in the automated decomposition, as expected, as *R* is the objective function of the minimisation in the FCP-based formulation.

By investigating the relationship between filament length and pairwise roughness, we can distinguish three regions (Fig. 3f): Long filaments typically correspond to actin bundles and exhibit small roughnesses (Fig. 3f_1_), the majority of filaments is shorter with comparable roughnesses (Fig. 3f_2_), and some typically short filaments consist of only one edge with roughness given by the edge weight itself (Fig. 3f_3_; cf. Eq. 1). The angular distribution of filaments indicates that the majority of filaments is aligned parallel to the cell axis (Fig. 3f, dashed grey line) which has been suggested to support longitudinal cell growth^63^,^64^. While these reports of longitudinal alignment of the actin cytoskeleton were based on manual or qualitative measurements, our approach facilitates fully automated quantification of the alignment of individual filaments. Our findings show that the length of a filament correlates with its average weight (Fig. 3g; Pearson correlation coefficient *c*_P_ > 0 and *p*-value *p*_P_ < 0.05), i.e., thicker actin bundles stretch across the cell while individual thinner actin filaments are more locally confined, as expected^18^,^65^.

Finally, we study filament convolutedness, given by the ratio of the length of a filament and the largest side of a bounding box enclosing the filament, used as a measure for the curvedness of a filament^65^. We find that the convolutedness is slightly negatively correlated with the filament length (Fig. 3i, red; *c*_P,conv_ < 0 and *p*_P,conv_ ≥ 0.05), in agreement with previous findings in *Arabidopsis thaliana* pollen grain^65^ and other plant species^66^. In contrast to the automated approach used here, the existing studies of filament convolutedness required manual segmentation which may be biased by the user. Generally, and more severely, using a bounding rectangle to compute the convolutedness of a filament is biased by the orientation of the filament with respect to the x- and y-axis of the image. Therefore, we use the maximal filament angle as a non-biased measure for the maximal, local curvedness of a filament. By investigating the relation between the maximal filament angle and filament length, we find a significant negative correlation (Fig. 3i, grey; *c*_P,angle_ < 0 and *p*_P,angle_ < 0.05). This negative correlation reflects the known increase in stiffness of actin bundles with increasing bundledness and length^67^,^68^. Thus, our approach provides a fast means to investigate this property for individual filaments in a cellular context without laborious manual filament identification.

To further extend these findings, we extract the cytoskeletal networks from 100 frames of a movie of a plant actin cytoskeleton (cf. Methods). For each frame, we compute the optimal filament covers and analyse the filaments. The additional data support our reported findings (Supplemental Material S8).

Moreover, we repeat our analyses of the robustness of our approach against incomplete knowledge of the underlying network structure or noisy edge weights for the cytoskeletal network (cf. discussion of Fig. 1; Supplemental Material S7). In our first scenario, the removal of increasing numbers of edges typically moderately decreases the accuracy of the obtained filament covers, i.e., their agreement with the manual assignment as measured by JI^1^. While the removal of some critical edges leads to a more severe decrease in accuracy, there exist edges whose removal leads to an increase in accuracy. On average, the removal of one additional edge decreases JI^1^ by around 0.002. Consequently, a loss of 10% of the cytoskeletal network’s *E* = 179 edges still yields JI^1^ ≈ 0.6 which is comparable to similarity values between different manual assignments (cf. Fig. 3d; cf. Supplemental Material S7). In our second scenario, the adding of Gaussian noise of increasing standard deviation to the edge weights similarly, as expected, decreases the accuracy of the obtained filament covers. However, this effect is moderate, i.e, increasing the standard deviation by 1% of the original edge weight decreases JI^1^ by less than 0.001. Adding noise with a standard deviation of 20% of the original edges weights still yields JI^1^ ≈ 0.6. As for the robustness analyses of the contrived network, for strong noise, JI^1^ tends to a constant, non-zero value which suggests that some information about the filament structure may be obtained solely from the network topology, irrespective of the edge weights (cf. discussion of Fig. 1; cf. Supplemental Material S7).

**Figure 4:**
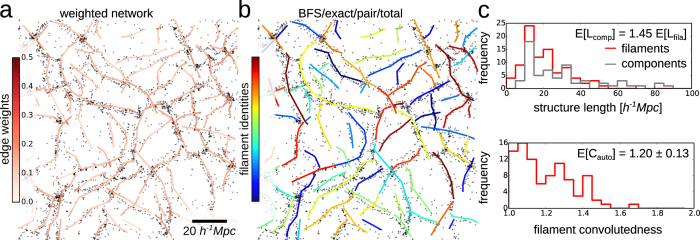
Filament covers and analyses of cosmic web. Image data from: Stoica *et al.*, A&A, 434, 423–432, 2005, reproduced with permission © ESO^14^. The cosmic web is decomposed automatically by solving the exact FCP (*exact*) for paths from a modified breadth-first search (*BFS*) and by minimising the total (*total*) pairwise filament roughness (*pair*). Distances are given in h^−1^Mpc, where currently h ≈ 0.7 is the dimensionless Hubble parameter^94^. (**a**) Overlay of simulated galaxy clusters and extracted network with colour-coded edge weights. (**b**) Automated decomposition of the cosmic web into galaxy filaments with colour-coded indices. (**c**) The length distribution of galaxy filaments exhibits a peak around 20h^−1^Mpc and levels off for larger lengths (upper panel, red). As a comparison, the distribution of the total lengths of the connected components, which was used as a measure of filament size in previous studies^14^, levels off more slowly and overestimates the average filament length by a factor of 1.45 (upper panel, grey; cf. average *L*-values). The distribution of the convolutedness of galaxy filaments suggests the prevalence of straight filaments and its average is comparable to that of the actin network (cf. 2i; cf. E[*C*] = 1.20 ± 0.13).

As a final example, we decompose the network of a simulated galaxy cluster (Fig. 4a) into individual galaxy filaments (Fig. 4b). The quantification of galaxy filaments may help to elucidate the acceleration of the universe^69^ and improve our understanding of large-scale structure formation^70^. Moreover, studies have revealed gravitational motion of galaxies along individual filaments^71^,^72^. Yet, previous studies focused on connected components of the cosmic web, and sought robust methods to identify individual filaments^14^,^70^. Our approach confirms the expected discrepancy between the lengths of the components (i.e., the sum of their edge lengths; Fig. 4c, upper panel, grey) and the length of individual filaments (Fig. 4c, upper panel, red; cf. average *L*-values). Moreover, the decomposition of the cosmic structures enables analyses of individual filament shapes. For example, the convolutedness which measures the curvedness of a filament shows small values (Fig. 4, lower panel), which are interestingly comparable to those found in the actin cytoskeleton (cf. Fig. 3i; cf. average *C*-values), indicating the prevalence of straight galaxy filaments.

In Table 2, we summarise the quality of the investigated decompositions of different filamentous networks and the options of the underlying FCP (cf. Supplemental Material S8 and S9 for analyses of additional filamentous networks that are not shown in the main text).

## Discussion

The decomposition of complex networks into meaningful substructures has facilitated network-based analyses of systems found in nature or designed by humans^73^,^74^,^75^. These natural and technical networks often embed filaments as basic building units. To enable deeper understanding of network systems with filamentous structure, it is therefore paramount to develop methods for accurate and feasible identification of the underlying filaments. In particular, the distinction between intra- and inter-filament connections enables a more detailed analysis of filamentous structures, including length statistics, spatial alignment, and bending of individual filaments. Such statistics may offer new insights, e.g., into the role of single actin or galaxy filaments in their cellular or cosmic network context, respectively (cf. Figs 3e–i and 4c).

Here, we proposed a robust optimisation approach to decompose any given weighted network into a set of smooth filaments comprising a filament cover. Since we demonstrated that the filament cover problem is intractable on general networks, we proposed, tested, and validated several alternative approximation schemes. The proposed approximation schemes are gauged at applications from different scientific fields in which filamentous structures naturally arise. We applied our optimisation-based approach on contrived test cases as well as biological and cosmic networks, and showed that it reliably identifies crossing, (non-) overlapping, and looped filaments in agreement with expert-based manual assignments.

Our approach offers a number of advantages over the existing alternatives:

1. The proposed optimisation approach can be applied to any weighted network. In particular, the approach can be readily applied to any network generated from two- or three-dimensional experimental image data typically gathered in biological studies and analyses of man-made systems (e.g.^51^,^76^,^77^,^78^), irrespective of the image source (e.g., light microscopy- or MRI-based). Thus, it may be used to study a variety of natural and technical filamentous structures in search for universal properties which go beyond the characterisation of geometric networks^79^.
2. Our approach facilitates the establishment of a link between the dynamics of individual filaments and the dynamics of the whole network. While the dynamics of individual filaments is guided by typically molecular, local processes, the behaviour of the entire filamentous structure incorporates and responds to stimuli across different scales. Therefore, the proposed approach provides the starting point towards network-oriented analysis of filaments. More specifically, the filament covers may even be used to track mobile filaments, as has been proposed for images of a few filaments using open contours^37^, providing a venue for fruitful applications of the method.
3. The different options of our approach, e.g., different measures of the filament roughness, enable flexible and intuitive customisation for different types of networks. For example, the filament roughness measure may include a penalty for filament bending in networks of straight, stiff filaments (such as microtubules^80^,^81^), or a penalty for length deviations in networks of filaments of mostly uniform length (such as synthetic polymers that are used, e.g., in drug delivery systems^82^,^83^).
4. At the same time, our approach to disentangle a given network is parsimonious, i.e., it has a strictly limited number of categorical options which allow testing of all possible combinations (4^2^ = 16 in total). In contrast, approaches which rely on multiple continuous parameters require data-specific and computationally expensive gauging of the parameters^38^,^41^. When compared to approaches which detect filaments directly from image data, however, the parsimony of our approach is counterbalanced by the parameter requirements of the preceding network extraction procedure.
5. Nevertheless, approaches that detect filaments directly from image data typically rely on local optimisation schemes and thus, e.g., on the order of filament initialisations and definitions of local filament properties^23^,^34^,^35^,^36^. In contrast, our approach offer the advantage that the decomposition into filaments is performed in a single optimisation step which holistically considers the global structure of both filaments and network.
6. Finally, since our approach replies on a general network representation, it may be applied also to networks obtained from other, e.g., open contour-based methods which often do not capture filament overlaps and result in fragmented filaments^22^,^23^. In a post-processing step, these fragments may be conveniently merged using our network-based approach (cf. Supplemental Material 10).

Yet, some caution is warranted:

1. The available options of the FCP yield different decompositions. We showed that paths sampled from a modified BFS enable more flexible and more accurate decompositions in comparison to paths sampled from RMSTs (cf. Fig. 1); in contrast to minimising the the average roughness, the minimisation of the total roughness favours longer filaments in better accordance with the manual assignments (cf. Fig. 1); moreover, since filament overlaps in biological systems may lead to an abrupt increase in apparent filament thickness, the proposed all-to-all filament roughness may be more suitable to study such situations than the pairwise filament roughness which favours filaments of slowly varying thickness. Therefore, the suitable choice of feasible and suitable options has to be further investigated. For example, for the actin cytoskeletal networks, it is not obvious if overlapping filaments should be preferred over non-overlapping filaments and if the pairwise roughness is a better measure of filament quality than the all-to-all roughness. Yet, such decision problems are innate not only to all automated decomposition algorithms, but also to the manual assignment based on which the performance is assessed. Thus, exploring different decomposition options by an expert in the field may hint at the right choice.
2. The quality of the filament cover clearly depends on the quality of the input network. To this end, several algorithms have been proposed for the extraction of various types of networks from image data with low error rates^23^,^33^,^38^,^39^,^40^,^84^. Moreover, we investigated different scenarios to test the robustness of our approach against incomplete knowledge of the underlying network structure as well as low signal-to-noise ratios and found that the accuracy of the filament cover is only moderately affected by these obstacles (cf. Supplemental Material S7).
3. Another issue are the computational requirements of the FCP. Although our proposed approximation scheme employs a modified BFS and a binary linear program which run fast on the tested networks, it may become infeasible for larger networks comprising more edges or nodes of larger degrees. Therefore, future efforts may focus on devising algorithms which approximate the FCP by employing local searches, i.e., without sampling a large number of paths for the proposed set cover-based approximation scheme.
4. Finally, we note that many polymers are not simple linear chains but branched tree-like structures^85^,^86^. Also many neurons may be naturally described as tree-like structures^87^,^88^. Our approach can be extended to account for these cases, thus, opening a new field of research. To this end, covering networks with more complex structures, such as stars^89^,^90^,^91^ or, more generally, trees^92^,^93^ may be employed. Due to intractability of these problems, investigation of approximation schemes like our set cover formulation will be needed. A central question will be the development of measures for the quality of a given star or tree cover.

In conclusion, by decomposing technically and biologically relevant filamentous structures into their constitutive filaments, our approach allows to see both the wood and the trees.

## Additional Information

**How to cite this article**: Breuer, D. and Nikoloski, Z. DeFiNe: an optimisation-based method for robust disentangling of filamentous networks. *Sci. Rep.* **5**, 18267; doi: 10.1038/srep18267 (2015).

## Supplementary Material

Supplementary Information

## Figures and Tables

**Table 1:** Overview of different approaches for disentangling filamentous networks.

Input	Method	Features					References
		curved filaments	filament-specific	intensity-based	automated	parsimonious	
image	texture filter	−	−	+	+	+	30
	linear programming	−	−	+	+	+	31
	rotating grid	−	+	+	+	+	32
	filament tracing	+	+	+	○	○	33, 34, 35
	filament tracking	+	+	+	+	○	36
	open contours	+	+	+	+	○	23,37
network	rule-based decomp.	+	+	−	○	○/−	38,41
	filament cover	+	+	+	+	○/+	current work

Two main classes of approaches to analyse the filamentous structure of networks can be distinguished, based on whether they operate on image data or on extracted networks. Irrespective of the class, the existing approaches vary in their capacity (+) or inability (−) to detect curved filaments, identify individual filaments, and to include information about the intensity/thickness of filaments. Further, the amount of manual user input as well as the number of parameters required by the algorithms can be feasible (+), laborious (−), or depends on the specific variant of the algorithm (○). For the network-based approaches, the number of required parameters may be different for the extraction of the network from image data and the consequent decomposition of the network into filaments (separated here by/).

**Table 2:** Quality of filament covers of artificial, biological, and cosmic networks in comparison to manual decompositions.

		Figure	Options	Similarity				
				VI	RI(≡RI^∞^)	JI(≡JI^∞^)	RI^1^	JI^1^
artificial	overlaps + loop	1	*BFS over pair tot*	0.792	1.000	1.000	1.000	1.000
	grid-like	S5b	*BFS exact pair tot*	0.889	0.962	0.742	0.941	0.872
neural	hippocampus	2	*BFS exact pair tot*	0.848	0.906	0.427	0.954	0.937
	retina	S5d	*BFS exact pair tot*	0.792	0.963	0.397	0.905	0.883
cytoskeletal	actin (FABD-labelled)	3	*BFS exact pair tot*	0.829	0.976	0.366	0.854	0.655
	actin (Lifeact-labelled)	S5f	*BFS exact pair tot*	0.530	0.929	0.193	0.838	0.701
cosmic	galaxy cluster (sparse)	4	*BFS exact pair tot*	no manual assignment				
	galaxy cluster (dense)	S5h	*BFS exact pair tot*	for comparison				

A given network is decomposed into filaments by solving the FCP with different options: The initial set of paths is obtained from a modified breadth-first search (*BFS*) or sampling of random minimum spanning trees (*RMST*), the filaments may overlap (*over*) or not (*exact*), a pairwise (*pair*) or all-to-all filament roughness measure (*all*) is used, and the total (*total*) or average (*avg*) roughness is minimised. The table displays the investigated filament covers with high similarity to the manual assignments.
