# Supplementary material for: DeFiNe: an optimisation-based method for robust disentangling of filamentous networks
Source: Sci Rep. 2015 Dec 15;5:18267. doi: 10.1038/srep18267 (PMC4678892; doi:10.1038/srep18267)
Supplement: Supplementary Information [file srep18267-s1.pdf]

DeFiNe: an optimisation-based method for robust disentangling of filamentous networks

Supplemental Material S1-S10

David Breuer[1,*] and Zoran Nikoloski[1]

[1]*Systems Biology and Mathematical Modeling, Max Planck Institute of Molecular Plant Physiology, Am Muehlenberg 1, 14476 Potsdam, Germany*

[*]*breuer@mpimp-golm.mpg.de*

SUPPLEMENTAL MATERIAL S1: MATHEMATICAL FORMULATION OF THE FILAMENT COVER PROBLEM

The structure of a filamentous network is described by a weighted geometric graph $G = (\mathcal{N}, \mathcal{E})$ with $N = |\mathcal{N}|$ nodes and $E = |\mathcal{E}|$ undirected, weighted edges. Edges represent filament segments and nodes represent their endpoints. The positions of the nodes are v_n, $n \in \mathcal{N}$, whereby, typically, $v_n \in \mathbb{R}^2$ or $v_n \in \mathbb{R}^3$ for networks extracted from image data. We focus on geometric networks because filaments are embedded in space, but our approach is readily applicable to non-geometric graphs. The edge weights are w_e, $e := (n_0, n_1) \in \mathcal{E}$ and $n_0, n_1 \in \mathcal{N}$.

To decompose the graph G into individual filaments it is natural to decompose it into paths, i.e., to solve a path cover problem (PCP). The PCP has been intensively studied on different types of graphs and with various restrictions (e.g. [95–100]). There are several potential routes (cf. [96] for an overview of the PCP for testing printed circuits): (1) We may either use node- or edge-paths, where a path $p = (a_{p,1}, \ldots, a_{p,P})$ is an ordered sequence of $P = |p|$ pairwise adjacent nodes ($a \in \mathcal{N}$) or edges ($a \in \mathcal{E}$), respectively, and $a_{p,i}$ denotes the i-th node or edge of filament p. (2) The paths may be either node-disjoint, edge-disjoint, or unrestricted. (3) The objective of the PCP may be either to obtain a cover of minimum cardinality or minimum weight.

For our purpose, the decomposition of a filamentous network into individual smooth filaments, it seems reasonable to look for an edge-path cover where each edge is covered by

(at least) one path and the total (or average) roughness is minimised. Edges that are covered by more than one path naturally correspond to filament overlaps. The minimisation of the average instead of the total roughness favours shorter paths which may be appropriate for some networks.

To define our filament cover problem (FCP) more rigorously, we introduce the roughness r_p of path p and the set $\mathcal{P}$ of all paths in G:

> Given a set $\mathcal{E}$ of edges and a set $\mathcal{P}$ of paths with roughnesses r_p, $p \in \mathcal{P}$:
>
> Find a subset $\mathcal{P}_{\text{fil}} \subseteq \mathcal{P}$ with minimal total (or average) roughness R such that each element in $\mathcal{E}$ is covered (at least) once.

The roughness measure r_p of a path p can be chosen arbitrarily and may involve, e.g., the edge weights or the edge alignments. An intuitive choice is the pairwise filament roughness of p (cf. Eq. 1),

$$r_{p,\text{pair}} = \begin{cases} (P-1)^{-1} \sum_{i=1}^{P-1} \left| w_{e_{p,i+1}} - w_{e_{p,i}} \right| & , P > 1 \\ w_{e_{p,1}} & , P = 1 \end{cases}, \tag{S1}$$

where $w_{e_{p,i}}$ denotes the weight of the i-th edge in filament p. The pairwise filament roughness is the average absolute value of the difference between weights of adjacent edges. It reflects the consistency of the edge weights along a filament which is typically smaller within than across filaments (but cf. Discussion). Moreover, if the path consists of a single edge we take its weight as a roughness measure. This choice increases the flexibility of the obtainable filament covers and is necessary to avoid a cover by only individual edges which contribute zero weight when weighted only according the first line in Eq. S1. Another measures for the quality of a filament is the all-to-all filament roughness (cf. Eq. 2)

$$r_{p,\text{all}} = \begin{cases} (P-1)^{-1} \max\limits_{i,j \in \{1,\ldots,P\}} \left| w_{e_{p,i}} - w_{e_{p,j}} \right| & , P > 1 \\ w_{e_{p,1}} & , P = 1 \end{cases}, \tag{S2}$$

which is the average maximal difference between any edge weights in a path p, and again the original weight of the edge is used for a path of length one. Taking into account that most filaments are only moderately bent, we may further wish to minimise the maximal filament

deflection angle between adjacent edges of a path p (cf. Eq. 3),

$$r_{p,\text{angle}} = \max_{i \in \{1,\ldots,P-1\}} \text{angle}\left(v_{e_{p,i+1,1}} - v_{e_{p,i+1,0}}, v_{e_{p,i,1}} - v_{e_{p,i,0}} \right) \tag{S3}$$

where $v_{e_{p,i,0}}$ and $v_{e_{p,i,1}}$ denote the positions of the start and end nodes of the i-th edge of filament p, respectively. Moreover, $\text{angle}\left(v, v'\right) := \arccos\left(\frac{v \cdot v'}{\sqrt{v \cdot v}\sqrt{v' \cdot v'}}\right)$ is the Euclidean angle of two vectors v and v' and $r_{p,\text{angle}} = 0°$ corresponds to perfectly straight alignment.

SUPPLEMENTAL MATERIAL S2: COMPUTATIONAL INTRACTABILITY OF THE FILAMENT COVER PROBLEM

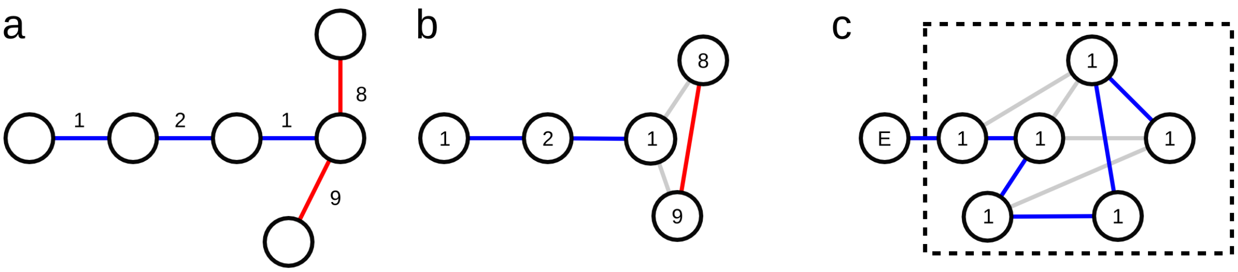

	total	average
pair	(1+1)/2 + (1)/1 = 2	2/2 = 1
all	(1)/2 + (1)/1 = 3/2	(3/2)/2 = 3/4

	total	average
pair	(0+0+...+0+(E-1))/(E-1) = 1	1/1 = 1
all	(E-1)/(E-1) = 1	1/1 = 1

Figure S1. **Proof of NP-hardness of the filament cover problem.** **(a)** Optimal filament cover of an exemplary (edge-weighted) graph. Table with cover roughnesses R for minimisation of total or average roughness and pairwise or all-to-all filament roughness measure, respectively. **(b)** Corresponding (node-weighted) line graph with equivalent path cover and the same roughness results as for the (edge-weighted) graph in (a). **(c)** Extension of an arbitrary graph with node weights 1 by a node of weight E. Here, finding a (node-weighted) path cover of roughness $R = (0 + 0 + \cdots + (E-1)) / (E-1) = 1$ or less is equivalent to finding a Hamiltonian path. This equivalence holds for covers minimising the total or average roughness of the cover and using the pairwise or all-to-all filament roughness measure (Eqs. S1 and S2), see table.

The FCP is difficult to solve. This is intuitively clear as the number of paths (let alone the number of path covers) increases rapidly with the number of nodes N. Even in planar

graphs, the number of closed paths visiting each node once was shown to increase at least exponentially with N [101, 102]. We show now that the FCP is NP-hard, even for planar, cubic graphs. Planar graphs can be drawn on a plane without crossing edges. They are of particular relevance since graphs that are generated from two-dimensional image data are planar by construction [40**?**]. Cubic graphs have only nodes of degree three. A proof of NP-hardness of a problem for planar, cubic graphs directly implies its NP-hardness on general graphs. The basic idea of a typical proof of computational complexity is as follows [103]: A problem of known complexity is selected. By providing a constructive transformation or reduction, a bijection between the known problem and the problem in question is established, i.e., any yes-instance of the decision-version of the known problem is mapped to a yes-instance of the decision-version of the problem of interest and analogously for the no-instances. This reduction proves that the two problems fall into the same class of computational complexity. Our proof is by reduction from the Hamiltonian path problem (HPP) on planar, cubic graphs which is known to be NP-complete [42]. The HPP asks, for a given graph, whether there is a node-path which visits each node exactly once.

First, we note that finding a filament cover on an edge-weighted graph G is equivalent to finding a node-path cover on its node-weighted line graph $L\left(G\right)$ (Fig. S1A and B). The line graph $L\left(G\right)$ of a graph G has a node of weight w_e for each edge e in G and edges connecting two nodes if the corresponding edges share a node in G.

Second, for a given line graph $L\left(G\right)$, we construct a graph such that finding a node-path cover of weight 1 or less is equivalent to solving the HPP. To that end, we add one edge with a terminal node to the line graph and set all original node-weights to 1 and the new node-weight to E (Fig. S1C). Then, only a Hamiltonian path ensures a minimal weight of $R = C^{-A} \sum_{i=1}^{C} r_{p_i} = \frac{1}{1} \frac{(0+\cdots+(E-1))}{(E-1)} = 1$, for both pairwise and all-to-all filament roughness $r_p = \{r_{p,\mathrm{pair}}, r_{p,\mathrm{all}}\}$ (cf. Eqs. S1 and S2) and both minimisation of total and average filament roughness, i.e., $A \in \{0, 1\}$.

Finally, we show that finding a Hamiltonian path on a line graph of a planar, cubic graph is NP-complete. It was shown that the HPP is NP-complete on general line graphs via a reduction from the HPP in cubic graphs [104]. This reduction remains valid when planar, cubic graphs are used instead of cubic graphs, for which NP-completeness of HPP is known [42]. Therefore, the decision version of the FCP is NP-complete and the FCP is NP-hard, as claimed. Since the FCP is NP-hard on planar, cubic graphs, it is (at least) NP-hard on

general graphs.

SUPPLEMENTAL MATERIAL S3: THE FILAMENT COVER PROBLEM ON TREES IS SOLVABLE IN POLYNOMIAL TIME

While we showed that the FCP is NP-hard on general and even planar, cubic graphs, it is solvable in polynomial time on trees. The polynomial algorithm outlined here is similar to those proposed to find an unrestricted node-path cover where each vertex may be included in multiple paths of minimum cardinality or minimum weight [99].

The basic idea is to assume that a certain path covering a certain edge is in the cover (in a tree, there are at most $N(N-1)/2 = \mathcal{O}(N^2)$ paths to choose from). Upon removal, the tree is split into potentially multiple forests (at most $\mathcal{O}(N)$), each tree of which is decomposed in the same way. The procedure is repeated for each edge (clearly $\mathcal{O}(N)$ in a tree). Thus, this results in a dynamic programming algorithm which has an overall polynomial time complexity of $\mathcal{O}(N^4)$.

The above procedure assumes non-overlapping paths and may be extended to limitedly overlapping paths. For the completely unrestricted case, there would be $\mathcal{O}\left(2^{\#\text{paths}}\right) = \mathcal{O}\left(2^{N^2}\right)$ combinations for covering a given edge to chose from in the first step, and the time complexity of the algorithm would be exponential. However, the problem remains polynomial if we allow only k-fold overlaps, $k = \mathcal{O}(1)$, i.e., each edge may be covered by at most k paths. In the first step of the above algorithm, a given edge may then be covered by at most $\mathcal{O}\left(\begin{pmatrix} N(N-1)/2 \\ k \end{pmatrix}\right) = \mathcal{O}\left(N^{2k}\right)$ edges and consequently the time complexity of the full algorithm is $\mathcal{O}\left(N^{2k+2}\right)$.

SUPPLEMENTAL MATERIAL S4: APPROXIMATION ALGORITHM FOR THE FILAMENT COVER PROBLEM

Since the FCP is NP-hard even on planar, cubic graphs, we need suitable approximation algorithms. In particular, the approximation algorithms should allow overlapping filaments as well as looped filaments. A natural choice seems to be the formulation of the FCP as a set cover problem (SCP) [53]:

Given an object set $\mathcal{U}$, called universe, and a set $\mathcal{S}$ of sets with costs c_s, $s \in \mathcal{S}$:

Find a subset $\mathcal{S}_{\text{set}} \subseteq \mathcal{S}$ with minimal total (or average) cost such that each element in $\mathcal{U}$ is covered (at least) once.

In our case, the universe corresponds to the set of edges of the given graph ($\mathcal{U} \hat{=} \mathcal{E}$), a set corresponds to a path ($s \hat{=} p$), the cost of a set corresponds to the roughness of a path ($c_s \hat{=} r_p$), and the set cover corresponds to the desired filament cover ($\mathcal{S}_{\text{set}} \hat{=} \mathcal{P}_{\text{fil}}$). We note, that this formulation of the SCP allows overlapping sets, $s \cap s' \neq \emptyset$, $s, s' \in \mathcal{S}$, which directly translates into overlapping filaments in our FCP. By requiring that each element in $\mathcal{U}$ is contained in $\mathcal{S}_{\text{set}}$ exactly once, we may exclude filament overlaps.

An open task is then the generation of a suitable set of paths ($\mathcal{S} \hat{=} \mathcal{P}$). Since for a general graph it is not feasible to find all paths $\mathcal{P}$ (cf. the motivation of the NP-hardness proof of the FCP above), we need to find a representative subset ,$\mathcal{P}'$, of paths. We propose two approaches: (1) We sample paths from $T = 100$ random minimal spanning trees (RMST) of G. To obtain a RMST, each edge is assigned a uniformly distributed random weight and the minimum spanning tree with respect to these weights is computed. Each tree has $N(N-1)/2$ non-trivial, undirected paths that we add to our set $\mathcal{P}'$. However, the paths in a tree cannot contain loops. (2) We perform a modified breadth-first search (BFS) on the nodes, store the generated paths, and stop the search for a path p when it violates a straightness criterion, e.g., $r_{p,\text{angle}} < 60°$ (cf. Eq. S3) which is used throughout the paper. We add all permitted paths to $\mathcal{P}'$. We note that for all real-world filamentous graphs, due to filament thickness, there are spatial constraints on the number of nodes of a graph as well as on the node degrees. Moreover, for the filamentous networks considered here, the radius of curvature of a filament is typically not much smaller than the region of interest. The number of loops is further reduced by the straightness criterion which eliminates paths with a small radius of curvature. Hence, the number of loops in the network is restricted and our heuristically modified BFS allows for loops and yields a representative set $\mathcal{P}'$ in reasonable time.

The SCP may be expressed as a binary fractional linear program [105], and we analogously write the FCP as

$$\begin{aligned} &\text{minimize}\, \frac{\sum_{p\in\mathcal{P}} r_{p,\text{pair}} x_p}{\left(\sum_{p\in\mathcal{P}} x_p\right)^A} \\ &\text{subject to} \sum_{p:e\in p} x_p \geq 1 \,\text{for all}\, e\in\mathcal{E} \\ &x_p \in \{0,1\} \,\text{ for all}\, p\in\mathcal{P}', \end{aligned} \tag{S4}$$

where in the first line $A \in \{0, 1\}$ determines whether the total or the average roughness is minimised. In the second line, equality holds for an exact cover. For $A = 0$, Eq. S4 is a binary linear program that may be solved using well-established and efficient algorithms [43, 44].

For $A = 1$, the fractional problem may be rewritten as a binary linear program as well [106, 107]. To that end, we introduce new variables $y = \left(\sum_{p\in\mathcal{P}} x_p\right)^{-1}$ and $z_p = x_p y$, $p \in \mathcal{P}'$. The latter expression is non-linear but may be replaced by a set of binary linear equations, yielding

$$\begin{aligned} &\text{minimize} \sum_{p\in\mathcal{P}} r_p z_p \\ &\text{subject to} \sum_{p:e\in p} z_p \geq y \,\text{for all}\, e\in\mathcal{E} \\ &\sum_{p\in\mathcal{P}} z_p = 1 \\ &y \geq 0 \\ &y - z_p \leq M - M x_p \\ &z_p \leq y \\ &z_p \leq M x_p \\ &z_p \geq 0 \\ &x_p \in \{0,1\} \,\text{ for all}\, p\in\mathcal{P}. \end{aligned} \tag{S5}$$

Here, M is a sufficiently large constant that needs to exceed any y (cf. the Big M method [108]). Since $y = \left(\sum_{p\in\mathcal{P}'} x_p\right)^{-1} \leq 1$ for the cover of any non-empty graph, we choose $M = 2$.

Thus, there are a number of options in our FCP: The input set of paths may be obtained by using a modified BFS or from sampling RMSTs or (denoted by either *BFS* or *RMST*). The filaments may overlap or not (*over*/*exact*). The objective of the FCP may be the

minimisation of the total or the average roughness (*total*/*avg*). The roughness of a filament may be measured by the pairwise or the all-to-all filament roughness (*pair*/*all*). Solutions of the FCP with different options are compared in the Results.

An implementation of the presented approximation schemes to the FCP with the described options is supplied as an open-source tool, "DeFiNe" (**De**composing **Fi**lamentous **Ne**tworks), under GLP3 at http://mathbiol.mpimp-golm.mpg.de/DeFiNe/. DeFiNe is programmed in Python [109] and employs the packages SciPy [110], NetworkX [111], and cvxopt [112] and PyGTK [113] for a simple and user-friendly graphical user interface. DeFiNe takes as input a weighted graph in the standard .gml file format [54] and outputs a standard .gml graph with filament identities stored as edge colours. Node coordinates may be included in the input file to enable the modified BFS that takes into account edge alignments. Furthermore, manual filament assignments may be included in the input file and the similarity with the automatically obtained filament cover is assessed as described below. In addition, DeFiNe generates a standard, human-readable .csv-table of various individual filament measures for custom analyses. The filamentous structure as well as the manual filament assignments shown in Fig. 1 are available as a .gml file under the above internet address for demonstration purposes.

SUPPLEMENTAL MATERIAL S5: EXTRACTION OF WEIGHTED NETWORKS FROM IMAGES

The procedure used to extract weighted networks from image data is similar to those proposed in [40, 41]: (1) The original grey-scale image are pre-processed to enhance the filamentous structures. Here, a vesselness filter with kernel width of 2 pixels was used for simplicity [114]. (2) In the filtered image, the filamentous structures are separated from the background by applying an adaptive median threshold with a block size of 49 pixels, whereby moderate variations of this size leave our findings largely unchanged. (3) The resultant binary image is skeletonised to obtain the filament centre lines [115]. (4) Then, the nodes of the network under construction are extracted as terminal points, branching points, or crossings of skeleton branches. (5) An edge is inserted between two nodes if they are directly connected via the skeleton. (6) Finally, the edges are weighted by integrating the intensity of the underlying original grey-scale image smoothed with a Gaussian filter with

a standard deviation of 5 pixels along the filament and taking its average per unit length of the filament. For the images of the simulated galaxy clusters, the structures obtained by a model-based filter from [14], Figure 6 left middle and bottom rows, are directly employed as binary images and the networks representations are obtained as described above.

SUPPLEMENTAL MATERIAL S6: QUALITY ASSESSMENT OF FILAMENT COVERS VIA STRUCTURE-AWARE PARTITION SIMILARITY MEASURES

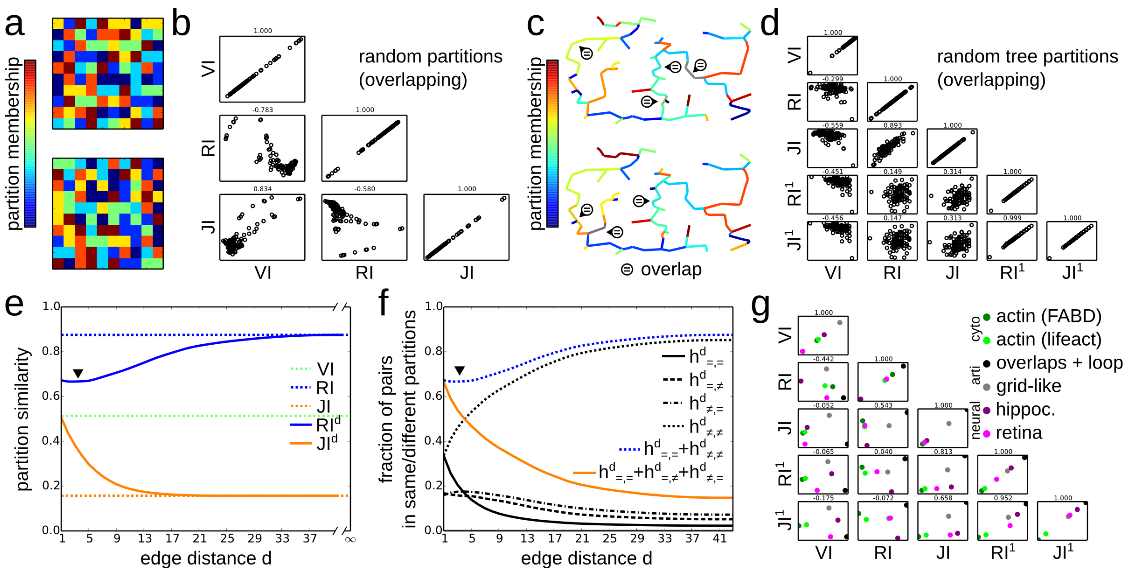

Figure S2. **Comparison of classical and extended partition similarity measures.** Analysis of 100×2 random partitions of sets of 100 numbers plus 10 duplicate ones into $5 - 10$ partitions (a-b). Analysis of a 100×2 path covers of Euclidean minimum spanning trees with 100 nodes distributed uniformly in the unit square, where the paths are drawn randomly and added if the overall overlap of paths is below 10 edges (c-f). Analysis of the similarities between the manual and automated decompositions of the networks studied in the paper (g). **(a)** Colour-representation of two exemplary random partitions as explained above. **(b)** The classical partition similarity measures VI, RI, and JI are not correlated (cf. Kendall rank correlation coefficients $\tau < 0.9$) and may lead to opposing conclusions for the similarity of different partitions. **(c)** Two exemplary random tree path covers with overlaps ($\ominus$). **(d)** The classical partition similarity measures VI, RI, and JI show no correlation among themselves (except for the pairing of RI and JI), nor with the structure-aware RI^1 and JI^1. In contrast, RI^1 and JI^1 are very strongly correlated (cf. $\tau > 0.9$) and yield consistent results for the similarity of different partitions. **(e)** Similarity of the partitions shown in (c) in dependence on maximal distance d between considered pairs of edges (cf. Fig. 3 for a detailed discussion). The RI^d shows a non-monotonous dependency on d (triangle). **(f)** This non-monotonicity of RI^d may be explained by the entries of the contingency table $h_{\times,\times'}$, $\times, \times' \in \{=, \neq\}$. For small distances d, the fraction of true positives (solid black, $h_{=,=}$) drops slower than the fraction of true negatives (dotted black, $h_{\neq,\neq}$) and for larger d, this trend is reversed. Hence, their sum (dashed blue) shows a minimum at intermediate distances d (triangle). In contrast, when summed up (solid yellow), the fast drop in the fraction of true positives dominates over the slightly non-monotony of the false positives and negatives. **(g)** For the investigated artificial and biological networks, the classical measures VI, RI, and JI yield partially opposing results on the similarity of the manual assignment and the automated decomposition (cf. $\tau < 0$). The structure-aware similarity measures RI^1 and JI^1 are strongly correlated and yield consistent results (cf. $\tau = 0.952$).

The extraction of the filamentous networks from image data enables comparison of the automated filament cover with manual filament assignments. Both, automated cover and manual assignment may be regarded as partitions (where we allow overlapping subsets as well). As measures for the similarity of the automated and manual partitions we use the variation of information,VI, the Jaccard index, JI, and the Rand index, RI, which are commonly used and were shown to estimate similarity reliably for distant and close partitions

alike [45–47]. For given partitions $\mathcal{C} = \{C_1, \ldots, \mathcal{C}_C\}$ and $\mathcal{C}^{'} = \left\{C_1^{'}, \ldots, \mathcal{C}_{C'}^{'}\right\}$, they are computed via

$$\begin{aligned} \mathrm{VI}\left(\mathcal{C}, \mathcal{C}^{'}\right) &= 1 + (U \log U)^{-1} \cdot \\ &\quad \cdot \sum_{i,j} g_{i,j} \left(\log\left(\frac{g_{i,j}}{g_{\cdot,j}}\right) + \log\left(\frac{g_{i,j}}{g_{i,\cdot}}\right)\right), \end{aligned} \tag{S6}$$

$$\mathrm{RI}\left(\mathcal{C}, \mathcal{C}^{'}\right) = \frac{h_{=,=} + h_{\neq,\neq}}{h_{=,=} + h_{=,\neq} + h_{\neq,=} + h_{\neq,\neq}}, \tag{S7}$$

$$\mathrm{JI}\left(\mathcal{C}, \mathcal{C}^{'}\right) = \frac{h_{=,=}}{h_{=,=} + h_{=,\neq} + h_{\neq,=}}, \tag{S8}$$

where $U = \sum_{i=1}^{C} |\mathcal{C}_i| = \sum_{j=1}^{C'} \left|\mathcal{C}_j^{'}\right|$, $g_{i,j} = \left|\mathcal{C}_i \cap \mathcal{C}_j^{'}\right|$, $g_{\cdot,j} = \sum_{i=1}^{C} g_{i,j}$, and $g_{i,\cdot} = \sum_{j=1}^{C'} g_{i,j}$. The contingency tables $h_{\times,\times'}$, $\times, \times^{'} \in \{=, \neq\}$, provide the numbers of edge pairs which are in the same or different sets in the two partitions, respectively, and is related to $g_{i,j}$ as shown in [57]. All measures are restricted to the unit interval with larger values reflecting higher similarity [58].

While these measures of partition similarity are widely used [46, 48], they pose some difficulties. The variation of information, VI, is only well-defined for disjoint partitions, which occur for non-overlapping filaments. While the Jaccard index, JI, and the Rand index, RI, cover intersecting partitions they may generally yield opposing results. We demonstrate this inconsistency by investigating two types of random partitionings: First, for 100 repetitions, we randomly partitioned 2 sets of 100 numbers and up to 10 duplicates (to simulate overlapping filaments) into $5 - 10$ random partitions (Fig. S2a). While VI and JI were correlated (Fig. S2b; cf. Kendall rank correlation coefficient $\tau > 0$), the other two combinations showed a strong negative correlation (cf. $\tau < 0$). Second, to study filament covers that resemble the decomposition of real filamentous networks more closely, we constructed a relative neighbourhood graph [116, 117] with 100 nodes uniformly distributed in the unit square and computed a random minimum spanning tree (Fig. S2c). For 100 repetitions of this procedure, we partitioned the resultant tree into filaments by choosing a path at random and adding it to the decomposition if the total overlap of any two paths already in the decomposition is below 10 edges (cf. $\ominus$ for overlaps). Again, the correlation among the classical similarity measures was poor or negative (Fig. S2d; except for the correlation between RI and JI; $|\tau| < 0.6$). Although other measure for the similarity of intersecting partitions have been proposed [118–120], we adhere to RI and JI for simplicity.

More severely, however, the above similarity measures do not take into account the structure of the graph G underlying the (edge-)partitions induced by the obtained filament covers. To date, we are only aware of structure-aware similarity measures for the comparison of partitions whose items are distributed in Euclidean space [121–123]. Yet, these approaches do not take into account the explicit graph structure of the partitions. To remedy this shortcoming, we introduce a suite of measures, the structure-aware Rand and Jaccard index, RI^d and JI^d, respectively. To that end, the contingency tables $h_{\times,\times'}$ in Eqs. S7 and S8 are replace by distance dependent $h^d_{\times,\times'}$,

$$\mathrm{RI}^d\left(\mathcal{C},\mathcal{C}'\right) = \frac{h^d_{=,=} + h^d_{\neq,\neq}}{h^d_{=,=} + h^d_{=,\neq} + h^d_{\neq,=} + h^d_{\neq,\neq}}, \tag{S9}$$

$$\mathrm{JI}^d\left(\mathcal{C},\mathcal{C}'\right) = \frac{h^d_{=,=}}{h^d_{=,=} + h^d_{=,\neq} + h^d_{\neq,=}} \tag{S10}$$

where $h^d_{\times,\times'}$, $\times,\times' \in \{=,\neq\}$, $d \in \mathbb{N}_{>0}$, count the number of edge pairs which are in the same or different sets in the two partitions, respectively, and which are separated by at most d nodes in G. More precisely, we define

$$h^d_{\times,\times'} = \Big\{ \#\left(e_0,e_1\right) \mid e_0 \in \mathcal{C}_i \cap \mathcal{C}'_{i'},\ e_1 \in \mathcal{C}_j \cap \mathcal{C}'_{j'}, \tag{S11}$$
$$\text{with}\, i \times j \,\text{and}\, i' \times' j' \,\text{and}\, D_{L(G)}\left(e_0,e_1\right) \leq d \Big\},$$

where $\#\left(e_0,e_1\right)$ is the number of edges $\left(e_0,e_1\right)$ and $D_{L(G)}\left(e_0,e_1\right)$ is the length of the shortest path between nodes in the line graph $L\left(G\right)$ of G corresponding to the edges e_0 and e_1. For example, $h^0_{=,=}$ counts the the number of adjacent edges which are in the same set in both partitions (local perspective). In contrast, $h^\infty_{\times,\times'} \equiv h_{\times,\times'}$ reproduce the original measures which do not take into account the positions of edges in the graph (global perspective).

To investigate the performance of our extended, structure-aware partition similarity measures, RI^d and JI^d, we apply them to the artificial graph-based random partitions described above (cf. Fig. S2c). Indeed, when considering the partition membership of neighbouring edges only, i.e., RI^1 and JI^1, the similarity measures yield very consistent results (Fig. S2d; cf. $\tau = 0.999$) in contrast to the lower correlation with the classical similarity measures (cf. $\tau < 0.9$). Investigating the dependency of RI^d and JI^d on the distance d for the tree filament covers shown in Fig. S2d, we find that RI and JI (Fig. S2e; dotted blue and yellow) over- and underestimate the partition similarity with respect to RI^1 and JI^1 (Fig. S2e; solid

blue and yellow). Furthermore, we find that RI^d is non-monotonic in d (Fig. S2e; cf. the black triangle). These errors in estimation are explained by the large fraction of false negatives ($h_{\neq,\neq}$) and the small fraction of true positives ($h_{=,=}$), respectively, which dominate for large distances d, i.e., the limit in which the graph structure is ignored (Fig. S2f; dotted black and solid black). Due to the differential increase/decrease of $h_{\neq,\neq}/h_{=,=}$, their combination and therefore RI^1 is non-monotonic (Fig. S2f; dashed blue). Finally, we observe opposing results of the classical partition similarity measures also for the filament covers of artificial and biological filamentous networks investigated in the main text, while our extended, structure-aware measures RI^1 and JI^1 provide consistent similarity results (Fig. S2g).

SUPPLEMENTAL MATERIAL S7: ROBUSTNESS OF FILAMENT COVERS AGAINST INCOMPLETE KNOWLEDGE OF UNDERLYING NETWORK STRUCTURE AND IMAGE NOISE

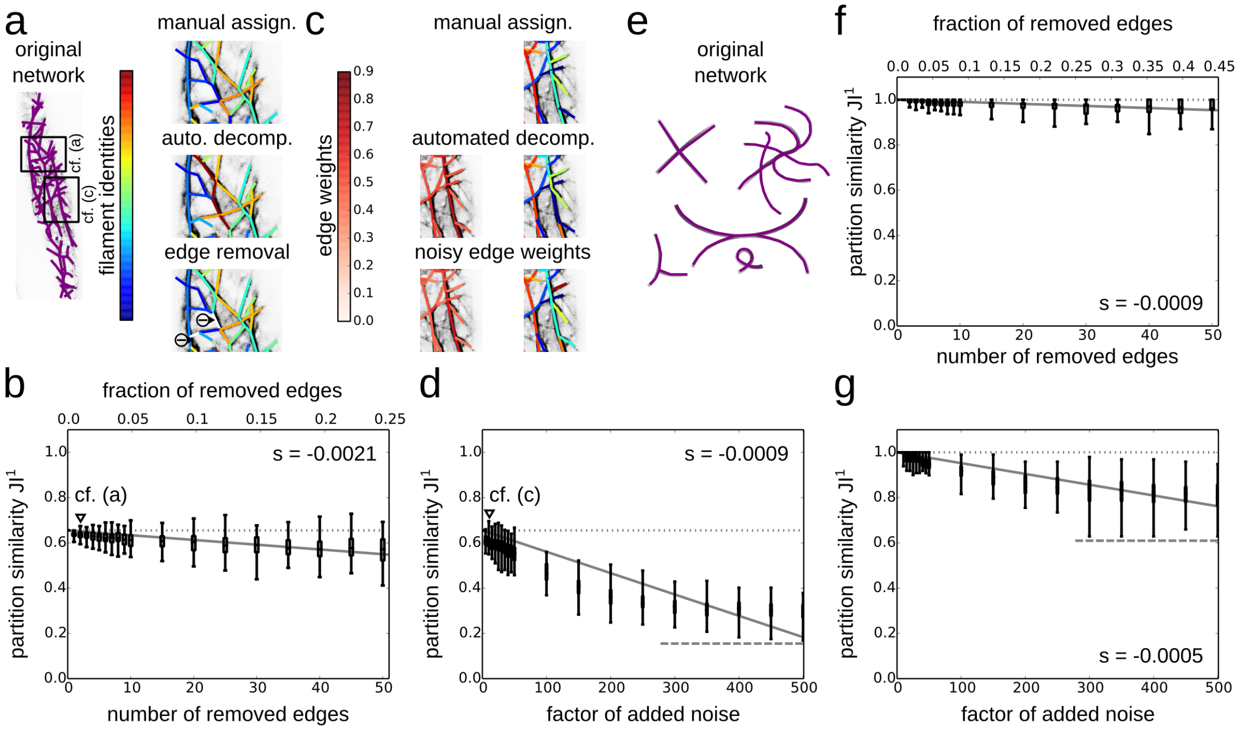

Figure S3. **Analyses of robustness of filament covers against incomplete knowledge of network and image noise.** A cytoskeletal and a contrived network are decomposed automatically by solving the FCP with options given in Fig. 3c and Fig. 1c, respectively. **(a)** Overlay of extracted actin network structure and original image data (left panel). Sections of cytoskeletal network with edge colours representing the manual assignment, the optimal filament cover obtained for the full, non-disrupted network, and the optimal filament cover after removal of two edges which are shown in white (right panels). **(b)** Similarity of manual filament assignment and automated filament covers after removal of increasing numbers of edges, measured by structure-aware Jaccard index JI^1. On average, JI^1 decreases with the number of removed edges as shown by a linear fit with slope $s = -0.0021$ (solid grey line). Occasionally, the removal of edges increases the accuracy of the filament cover above the accuracy of the original solution (dotted grey line and triangle; cf. panel (a)). **(c)** Sections of cytoskeletal network with edge colours representing the original edge weights and the edge weights after adding Gaussian noise (left panels). Sections of cytoskeletal network with edge colours representing the manual assignment, the optimal filament cover obtained for the full, non-disrupted network, and the optimal filament cover after adding Gaussian noise (right panels). **(d)** Similarity JI^1 of manual filament assignment and automated filament covers after adding Gaussian noise. On average, JI^1 decreases with increasing noise factor as shown by a linear fit with slope $s = -0.0009$ (solid grey line). Occasionally, the noisy edge weights lead to an increase in accuracy of the filament cover above the accuracy of the original solution (dotted grey line and triangle; cf. panel (c)). The decrease levels off for large noise factors and JI^1 approaches a constant value (dashed grey line). **(e)** Overlay of extracted contrived network structure and original image data. **(f)** Results for the contrived network analogue to those presented for the cytoskeletal network in panel (b). The average change in JI^1 per removed edge is captured by a linear fit with slope $s = -0.0009$. **(g)** Results for the contrived network analogue to those presented for the cytoskeletal network in panel (f). The average change in JI^1 per unit increase in the noise factor is captured by a linear fit with slope $s = -0.0005$.

Our approach enables accurate decomposition of a given filamentous network into its constitutive filaments (cf. Results). However, the preceding extraction of the network from image data is often non-trivial (cf. Methods). Therefore, to assess the robustness of our approach, we test how the accuracy of our filament decomposition is affected (1) by incomplete

knowledge of the true underlying network structure and (2) by image noise which affects the edge weights of the extracted network. We perform these analyses for the actin cytoskeleton shown in Fig. 3 (Fig. S3a, left panel) and the contrived network shown in Fig. 1 (Fig. S3e).

(1) First, we start from the original, weighted network and randomly remove one of the E edges to model erroneous segmentation. For the disrupted network, we recompute the optimal filament cover (with the same options as in Figs. 1c and 3c, respectively) and calculate its agreement with the original manual segmentation (measured by the structure-aware Jaccard index JI^1; the removed edge is assigned a dummy label). We repeat the procedure for E networks from which a single, randomly chosen edge has been removed. Next, we repeat the procedure for E networks from which a randomly chosen double of edges has been removed. We then proceed with triplets, quartets, and so on up to subsets of 50 randomly chosen edges.

As expected, the removal of increasing numbers of edges typically decreases the agreement of the automated filament cover with the manual assignment for the cytoskeletal as well as the contrived network (Fig. S3b and f). For both networks, however, the decrease is slow and JI^1 increases only by around 0.002 per removed edge (cf. Fig. S3b and f, solid grey line indicates linear fit). Interestingly, for the actin cytoskeleton, the removal of certain edges may even increase the accuracy of the filament cover (Fig. S3a, right panels show manual filament assignment and automated filament cover the original network, and an exemplary filament cover obtained after the removal of two edges, coloured white here, which improves the agreement with the manual assignment; cf. Fig. S3b, dotted grey line and triangle).

(2) Second, we simulate image noise by adding centred Gaussian noise Δw to the edge weights of the original network with

$$\mathrm{E}\left[\Delta w\right] = 0, \tag{S12}$$

$$\mathrm{Sd}\left[\Delta \mathrm{w}\right] = \left(1 + \frac{f}{100}\right) w. \tag{S13}$$

We normalise the standard deviation of the added noise by the original edge weights to avoid extreme fluctuations, and f is referred to as noise factor. For each noise factor, we construct 100 networks, recompute the optimal filament covers, and measure their agreement with the manual filament assignment, as in the first scenario above.

For both the contrived and the cytoskeletal network, the accuracy of the filament cover decreases with increasing noise, as expected (Fig. S3d and g). However, this decrease in

accuracy is slow and JI^1 decreases by less than 0.001 when increasing the standard deviation of the noise by 1% of the original edge weights, i.e., when increasing the noise factor by one (cf. Fig. S3d and g, solid grey lines indicate linear fits). We note that with increasing edge noise the accuracy of the filament cover approaches a constant, non-zero JI^1 which reflects that some information about the filament structure maybe obtained from the topology of the network alone, irrespective of the edge weights (cf. Fig. S3d and g, dashed grey lines).

SUPPLEMENTAL MATERIAL S8: FILAMENT ANALYSIS FOR NETWORKS EXTRACTED FROM MOVIE OF PLANT ACTIN CYTOSKELETON

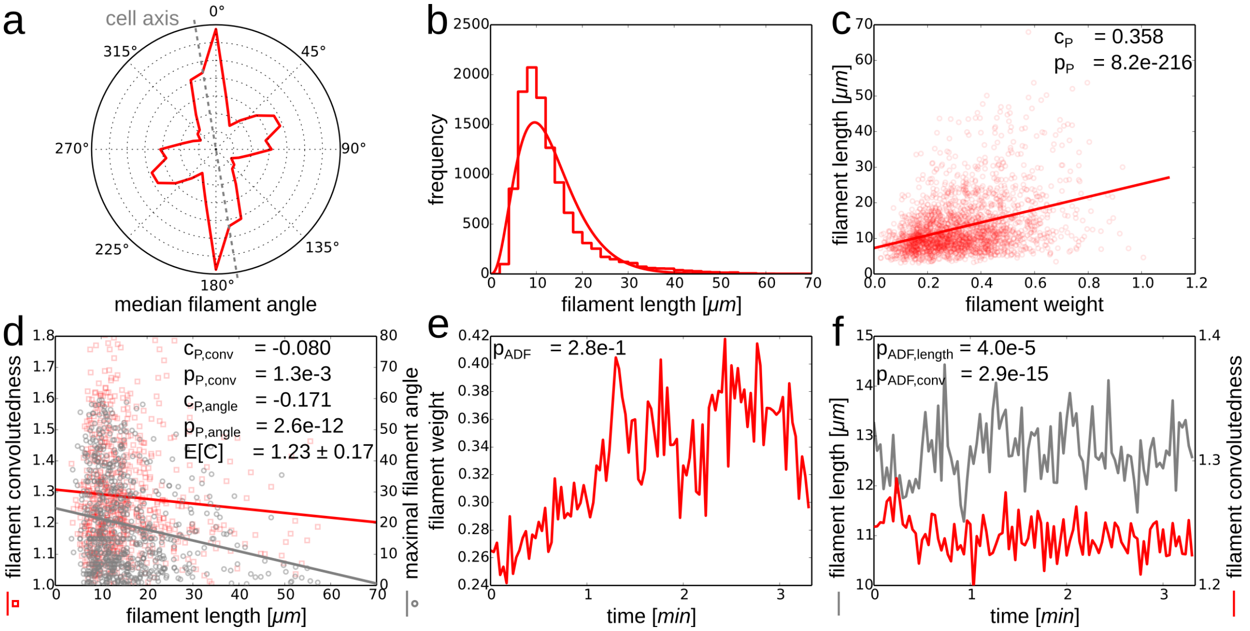

Figure S4. **Filament analyses of 100 cytoskeletal networks.** Results from filament decompositions of 100 cytoskeletal networks extracted from a movie of a plant cytoskeleton over 200 s. The cytoskeletal networks are decomposed automatically by solving the exact FCP (*exact*) for paths from a modified breadth-first search (*BFS*) and by minimising the total (*total*) pairwise filament roughness (*pair*; cf. Fig. 3). **(a)** The distribution of median filament angles shows that the majority of filaments is aligned parallel to the cell axis (grey dashed line). **(b)** Filament lengths (bars) follows a gamma distribution (line shows maximum likelihood fit). **(c)** Filament length correlates with filament weight (cf. linear regression and Pearson correlation coefficient $c_P > 0$ and p-value $p_{\mathrm{P}} < 0.05$) **(d)** Scatter plot of filament convolutedness versus filament length shows a negative correlation (cf. red squares, $c_{P,\mathrm{conv}} < 0$, and $p_{\mathrm{P,conv}} < 0.05$) with an average convolutedness of $\mathrm{E}\left[C\right] = 1.23 \pm 0.17$. The maximum filament angle correlates negatively with the filament length (cf. grey circles, $c_{P,\mathrm{angle}} < 0$, and $p_{\mathrm{P,angle}} < 0.05$), indicating that longer (and thicker, cf. (c)) filaments are less curved. **(e)** Time series of average filament weight over 200 s shows large fluctuations and is non-stationary (cf. augmented Dickey-Fuller test p-value $p_{\mathrm{ADF}} \geq 0.05$). **(f)** Time series of filament length and convolutedness are stationary over the recording period (cf. $p_{\mathrm{ADF,length}} < 0.05$ and $p_{\mathrm{ADF,conv}} < 0.05$).

To further strengthen our statistical analyses of cytoskeletal actin filaments (cf. Fig. 3), we investigate a complete movie of a plant cytoskeleton of 100 frames over 200 s (cf. Methods for details). For each frame, we extract a weighted network representation of the cytoskeleton as described above (cf. Methods for details) and solve the FCP with options described in Fig. 3, i.e., we solve the exact FCP (*exact*) for paths from a modified breadth-first search (*BFS*) and by minimising the total (*total*) pairwise filament roughness (*pair*). Analysis of various properties of the automatically obtained filaments confirms our findings in Fig. 3: The filaments show a preferential alignment parallel to the cell axis throughout the movie (Fig. S4a). The distribution of filament lengths, pooled across the duration of the movie, confirms the reported gamma distribution (Fig. S4b; maximal likelihood fits of normal, Weibull, and Rayleigh distributions yield higher values for the Akaike information criterion [60]). Filament length is correlated with filament weight, i.e., longer filaments are typically thicker (Fig. S4c; Pearson correlation p-value $p_{\mathrm{P}} < 0.05$). Moreover, the correlation between different measures of filament curvedness, i.e., the filament bending and the maximal filament

angle, are consistently negatively correlated with the filament length (Fig. S4d; $p_{\mathrm{P}} < 0.05$).

In addition to these previously analysed features of filamental organisation, we study the course of different filament properties over time: The average filament weight shows large fluctuations and is non-stationary over the recording period (Fig. S4e; cf. augmented Dickey-Fuller test p-value $p_{\mathrm{ADF}} \geq 0.05$). This non-stationarity suggests substantial changes in the prevalence of fine actin filament and thick bundles, respectively, and prompts further investigations. However, we found that the average filament length as well as the average filament bending remain stationary over the course of 200 s (Fig. S4e; cf. $p_{\mathrm{ADF,length}} < 0.05$ and $p_{\mathrm{ADF,conv}} < 0.05$). Since the length distribution of filaments tunes the mechanical properties of filamentous networks [124, 125], this stationarity of the average filament length may be of immediate biological relevance. The stationarity of the average filament bending may be a direct consequence of the roughly constant filament length distribution (cf. Fig. S4d) in combination with the resultant physical constraints of actin filament length on filament bending.

SUPPLEMENTAL MATERIAL S9: OVERVIEW OF DIFFERENT STAGES OF FILAMENT DECOMPOSITION OF ARTIFICIAL, BIOLOGICAL, AND COSMIC NETWORKS

We test our method of decomposing a given weighted network into filaments by solving the FCP for different filamentous networks. In addition to the four networks presented in the main text and the 100 frames analysed in Supplemental Material S8, we investigate four more networks of different types and show the different stages of our analysis. Starting from grey-scale image data of contrived, neural, cytoskeletal and cosmic network structures (Fig. S5, 1st column), we pre-process the images to obtain a binary representation of the filament centre lines (Fig. S5, 2nd column), and extract a weighted network representation as described in the Methods (Fig. S5, 3rd column). For the contrived and biological and the cosmic networks, we manually assign filament identities and compute the connected components, respectively (Fig. S5, 4th column). Finally, we decompose the networks into filaments by solving the FCP with different options (Fig. S5, 5th column). For the first contrived network, we allow overlapping filaments (Fig. S5a) while for the second, grid-like contrived network (Fig. S5b), the neural networks (Fig. S5c and d), the cytoskeletal networks

(Fig. S5e and f), and the cosmic webs (Fig. S5g and h), we obtain exact filament covers with options described in Fig. 1e. The agreement of manual assignments and automated filament decompositions of the studied networks is measured by the classical and the structure-aware Jaccard indices JI and JI^1 and shows good agreement (JI^1 close to 1, and cf. discussion of Fig. 3d) despite occasional over- (cf. $\oplus$) or under-segmentation (cf. $\ominus$) of filaments.

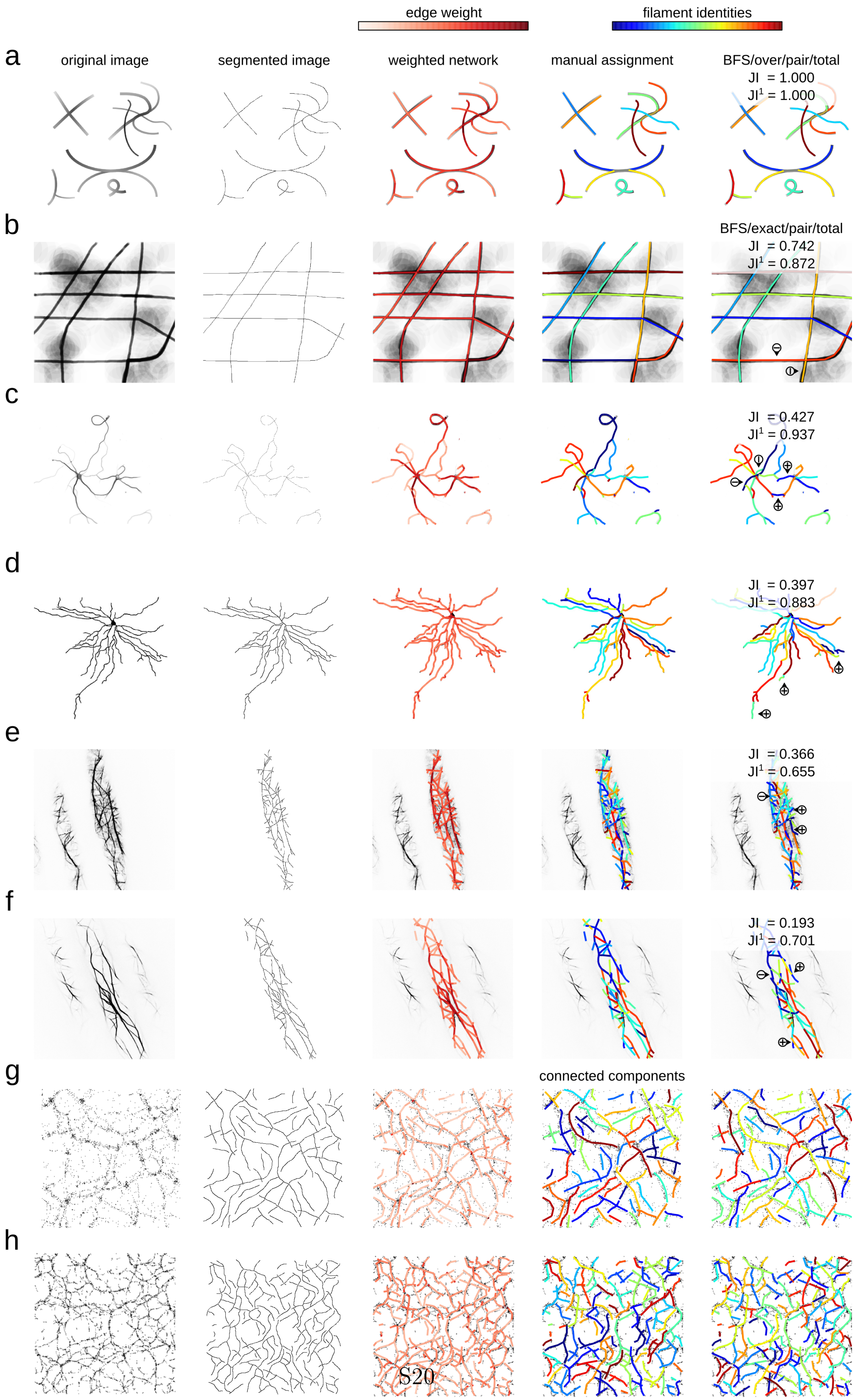

edge weight
filament identities
a
original image
segmented image
weighted network
manual assignment
BFS/over/pair/total
JI = 1.000
JI1 = 1.000
b
BFS/exact/pair/total
JI = 0.742
JI1 = 0.872
c
JI = 0.427
JI1 = 0.937
d
JI = 0.397
JI1 = 0.883
e
JI = 0.366
JI1 = 0.655
f
JI = 0.193
JI1 = 0.701
g
connected components
h

Figure S5. **Overview of studied networks, manual assignments, and filament covers obtained from solving the FCP.** Original grey-scale image data (1st column), binary images of filament centre lines (2nd column), extracted networks with colour-coded edge weights (3rd column), manual filament assignments of contrived and biological networks and connected connected components of cosmic networks, respectively (4th column), and automatically obtained filament covers (5th column). Agreement between manual decompositions and automated filament cover is quantified by a number of measures (cf., e.g., Methods and Fig. 2), here the classical and the structure-aware Jaccard indices JI and JI^1 are shown. **(a)** Contrived network with crossing and overlapping filaments and a loop (cf. Fig. 1). **(b)** For a contrived, grid-like network, the automated decomposition correctly detects most of the filaments (JI^1 close to 1). Only the filament in the bottom right corner with a kink is over-segmented (⊕) because the curvature restriction of the initial paths does not allow such large angles (about 90° here, cf. Eq. S3). **(c)** Neural network of hippocamal neuron (cf. Fig. 2). **(d)** The decomposition of the network of a retinal ganglion cell shows good agreement with the manual results (JI^1 close to 1). A few filaments are over-segmented (⊕), e.g., due to kinks in the filaments that are not captured by the initial set of paths (cf. the centre ⊕). **(e)** Cytoskeletal network of actin filaments (cf. Fig. 3). **(f)** For the actin network extracted from the confocal recording of a Lifeact-labelled cytoskeleton, the automated partitions agrees well with the manual results (JI^1 close to 1, and cf. discussion of Fig. 3d). A few examples of over- and under-segmentation (⊖) are marked. **(g)** Cosmic web of galaxies (cf. Fig. 4). **(h)** The dense web of simulated galaxies consists of many connected components that are further decomposed into filaments (cf. Fig. 4 for a discussion). Image data for panels (g) and (h) from: Stoica et al., A&A, 434, 423-432, 2005, reproduced with permission © ESO.

SUPPLEMENTAL MATERIAL S10: OPEN CONTOUR-BASED FILAMENT DECOMPOSITION AND FILAMENT COVER-BASED POST-PROCESSING

Finally, we demonstrate how our filament cover-based approach may be used to post-process and improve filament decompositions obtained from other, e.g., open contour-based approaches. For the demonstration, we select SOAX [23], a fully automated, stretching open active contour-based approach which is available as an open-source software tool to extract a network-like representation (i.e., coordinates of filament centre lines as well as junctions

are provided) from image data. As a test case, we study the contrived filamentous structure investigated in Fig. 1. For a fair comparison of our and the open contour-based approach, we apply SOAX to the pre-processed and segmented image data (cf. Methods and Fig. S6a, second panel) to which we further apply a Gaussian filter of unit standard deviation to obtain smooth intensity gradients required by the algorithm. SOAX is run using the default parameters and the resulting filament identities are manually assigned to match those of the manual solution (Fig. S6b). To quantify the quality of the decomposition, we manually assign filament identities in our original network representation (cf. Fig. S6a, third panel) according to the open contour-based result (cf. Fig. S6b) and compare the result to the manual assignment (cf. Fig. S6a, fourth panel). The structure-aware Jaccard index $\mathrm{JI}^1 = 0.938$ is close to 1 and indicates good agreement between open-contour based decomposition and manual filament assignment. We note that some junctions/nodes obtained from SOAX are split in two in comparison to our extracted networks (cf. intersecting $\bigcirc$).

Moreover severely, some filaments are over-segmented and thus fragmented (cf. $\oplus$), especially overlapping filaments which are not captured in the open contour-based approach (cf. $\ominus$). To remedy this shortcoming, we apply our filament cover-based approach to post-process the open contour-based decomposition and merge over-segmented filament fragments. To this end, we convert the open contour-based filament representation into a weighted network, where edge weights represent average filament segment intensities as before (cf. Methods and Fig. S6c). As before, a collection of paths $\mathcal{P}'$ is sampled using a breadth-first search (*BFS*) and their pairwise roughness values r_p, $p \in \mathcal{P}'$, are computed according to Eq. S1 (*pair*). Then, to take into account the initial open contour-based filament decomposition $\mathcal{F}$ as a starting point, in which certain edges have already been assigned to the a given filament, we modify the roughness values of the sampled paths: For each initial filament or fragment that is fully contained within a sampled path, the roughness of that path is decreased by a large value, $R_{\text{filament}} = 10^4$, which is larger than any r_p to favour the inclusion of these filaments or fragments in the optimal filament cover. Since the subtraction of R_{filament} yields negative roughness values which would lead to the inclusion of all these paths, we add another, even larger constant $R_{\text{offset}} = 10^8 > R_{\text{filament}}$ to all roughness values,

i.e.,

$$r_p' = r_p - \sum_{\substack{f \in \mathcal{F} \\ f \subset p}} R_{\text{filament}} + R_{\text{offset}}. \tag{S14}$$

For these modified roughness values r_p', we solve the FCP by minimising the total roughness (*total*) and allowing for overlaps (*over*; Fig. S6d). The resulting post-processed filament decomposition merges several filament fragments which were over-segmented by the open-contour based approach and shows very good agreement of JI $=$ 0.776 and JI^1 $=$ 1.000 with the manual filament assignment. Interestingly, in this decomposition, parts of two filaments are interchanged (cf. ⊕) as in Fig. 1f for different FCP options. In conclusion, for any approach that detects filaments from image data and yields a weighted network representation, our filament cover-based approach may provide a helpful means to further post-process and enhance the accuracy of the obtained filament decomposition.

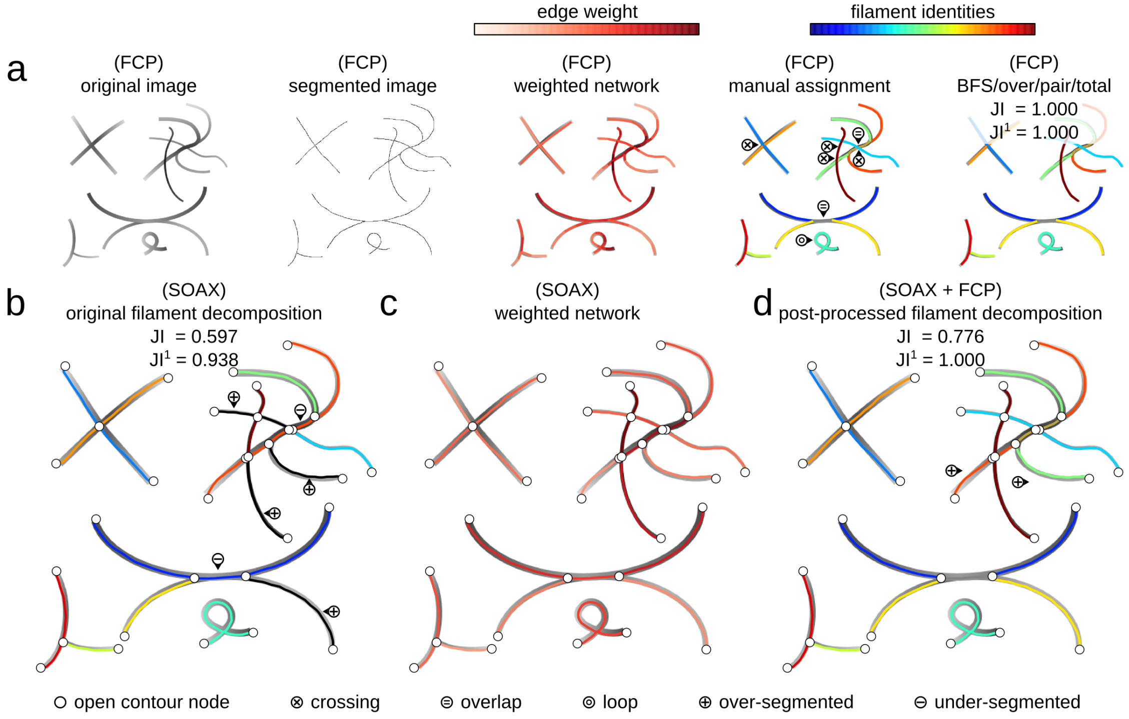

Figure S6. **Open contour-based filament detection and filament cover-based post-processing.** **(a)** Different stages of our filament cover problem (FCP)-based analysis for a contrived filament structure, from original image to segmented filament centre lines and weighted network representation, manual filament assignment and automated solution (cf. Fig. S5a for further explanations). **(b)** Filaments and junctions (cf. circles) identified from the segmented filament centre line image using SOAX, an stretching open active contour-based approach [23]. Colour-coded filament identities were manually assigned to match those of the manual solution in (a) and excess filament fragments were coloured black. While the agreement with the manual solution is good (JI^1 close to 1), some filaments are over-segmented (cf. $\oplus$) and thus fragmented, especially at locations of filament overlaps (cf. $\ominus$). **(c)** Weighted network representation of the contrived filamentous structure obtained from SOAX. **(d)** Using the filament assignments from SOAX in (b) as a starting point, our filament cover-based approach is used to post-process the filament decomposition, which merges broken filament fragments and improves the agreement with the manual solution ($\mathrm{JI}^1 = 1$).

---

[1] Stamm, A. J. *et al.* *Wood and cellulose science* (Ronald Press Co, New York, 1964), 1 edn.

[2] Klemm, D., Heublein, B., Fink, H.-P. & Bohn, A. Cellulose: fascinating biopolymer and sustainable raw material. *Angew Chem Int Ed Engl* **44**, 3358–3393 (2005).

[3] Shih, Y.-L. & Rothfield, L. The bacterial cytoskeleton. *Microbiol Mol Biol Rev* **70**, 729–754 (2006).

[4] Liu, B. *The plant cytoskeleton* (Springer, New York, 2010), 2 edn.

[5] Wickstead, B. & Gull, K. The evolution of the cytoskeleton. *J Cell Biol* **194**, 513–525 (2011).

[6] Braitenberg, V. & Schüz, A. *Cortex: statistics and geometry of neuronal connectivity* (Springer, Berlin, 1998), 1 edn.

[7] Lichtman, J. W., Livet, J. & Sanes, J. R. A technicolour approach to the connectome. *Nat Rev Neurosci* **9**, 417–422 (2008).

[8] Zhu, J., Ingram, P. A., Benfey, P. N. & Elich, T. From lab to field, new approaches to phenotyping root system architecture. *Curr Opin Plant Biol* **14**, 310–317 (2011).

[9] Galkovskyi, T. *et al.* GiA roots: software for the high throughput analysis of plant root system architecture. *BMC Plant Biol* **12**, 116 (2012).

[10] Lobet, G. *et al.* Root System Markup Language: toward a unified root architecture description language. *Plant Physiol* 114.253625 (2015).

[11] Gibson, S. & Fan, Y. Coronal prominence structure and dynamics: a magnetic flux rope interpretation. *J Geophys Res-Space* **111** (2006).

[12] Mackay, D., Karpen, J., Ballester, J., Schmieder, B. & Aulanier, G. Physics of solar prominences: II. magnetic structure and dynamics. *Space Sci Rev* **151**, 333–399 (2010).

[13] Bond, J., Kofman, L. & Pogosyan, D. How filaments of galaxies are woven into the cosmic web. *Nature* **380**, 603–606 (1996).

[14] Stoica, R. S., Martinez, V. J., Mateu, J. & Saar, E. Detection of cosmic filaments using the Candy model. *Astron Astrophys* **434**, 423–432 (2005).

[15] Bond, N. A., Strauss, M. A. & Cen, R. Crawling the cosmic network: identifying and quantifying filamentary structure. *Mon Not R Astron Soc* **409**, 156–168 (2010).

[16] Tully, R. B., Courtois, H., Hoffman, Y. & Pomarède, D. The Laniakea supercluster of galaxies. *Nature* **513**, 71–73 (2014).

[17] Moon, R. J., Martini, A., Nairn, J., Simonsen, J. & Youngblood, J. Cellulose nanomaterials review: structure, properties and nanocomposites. *Chem Soc Rev* **40**, 3941–3994 (2011).

[18] Akkerman, M., Overdijk, E. J., Schel, J. H., Emons, A. M. C. & Ketelaar, T. Golgi body motility in the plant cell cortex correlates with actin cytoskeleton organization. *Plant Cell Physiol* **52**, 1844–1855 (2011).

[19] Bálint, S., Verdeny Vilanova, I., Sandoval Álvarez, A. & Lakadamyali, M. Correlative live-cell and superresolution microscopy reveals cargo transport dynamics at microtubule intersections. *Proc Natl Acad Sci* **110**, 3375–3380 (2013).

[20] Kandel, E., Schwartz, J. & Jessell, T. *Principles of Neural Science* (McGraw-Hill, New York, 2000), 4 edn.

[21] Sporns, O., Tononi, G. & Kötter, R. The human connectome: a structural description of the human brain. *Plos Comput Biol* **1**, e42 (2005).

[22] Xu, T., Vavylonis, D. & Huang, X. 3D actin network centerline extraction with multiple active contours. *Med Image Anal* **18**, 272–284 (2014).

[23] Xu, T. *et al.* SOAX: a software for quantification of 3D biopolymer networks. *Sci Rep* **5**, 9081 (2015).

[24] Kumar, S. *et al.* Viscoelastic retraction of single living stress fibers and its impact on cell

shape, cytoskeletal organization, and extracellular matrix mechanics. *Biophys J* **90**, 3762–3773 (2006).

[25] Bausch, A. & Kroy, K. A bottom-up approach to cell mechanics. *Nat Phys* **2**, 231–238 (2006).

[26] Lu, L., Oswald, S. J., Ngu, H. & Yin, F. C.-P. Mechanical properties of actin stress fibers in living cells. *Biophys J* **95**, 6060–6071 (2008).

[27] Osunbayo, O. *et al.* Cargo transport at microtubule crossings: evidence for prolonged tug-of-war between kinesin motors. *Biophys J* **108**, 1480–1483 (2015).

[28] Eccles, J. C. The synapse: from electrical to chemical transmission. *Annu Rev Neurosci* **5**, 325–339 (1982).

[29] Bennett, M. *Electrical transmission: a functional analysis and comparison to chemical transmission*, 357–416 (Handbook of Physiology, Bethesda, 1977), 1 edn.

[30] Boudaoud, A. *et al.* FibrilTool, an ImageJ plug-in to quantify fibrillar structures in raw microscopy images. *Nat Protoc* **9**, 457–463 (2014).

[31] Wood, S. T., Dean, B. C. & Dean, D. A linear programming approach to reconstructing subcellular structures from confocal images for automated generation of representative 3D cellular models. *Med Image Anal* **17**, 337–347 (2013).

[32] Jacques, E. *et al.* Microfilament Analyzer, an image analysis tool for quantifying fibrillar orientation, reveals changes in microtubule organization during gravitropism. *Plant J* **74**, 1045–1058 (2013).

[33] Cohen, A. R., Roysam, B. & Turner, J. N. Automated tracing and volume measurements of neurons from 3D confocal fluorescence microscopy data. *J Microsc* **173**, 103–114 (1994).

[34] Meijering, E. Neuron tracing in perspective. *Cytom Part A* **77**, 693–704 (2010).

[35] Peng, H. *et al.* BigNeuron: large-scale 3D neuron reconstruction from optical microscopy images. *Neuron* **87**, 252–256 (2015).

[36] Mayerich, D. M. & Keyser, J. Filament tracking and encoding for complex biological networks. In *Proc 2008 ACM Symp Solid Phys Model*, SPM '08, 353–358 (ACM, New York, 2008).

[37] Smith, M. B. *et al.* Segmentation and tracking of cytoskeletal filaments using open active contours. *Cytoskeleton* **67**, 693–705 (2010).

[38] Leandro, J. J. G., Cesar-Jr, R. M. & Costa, L. d. Automatic contour extraction from 2D neuron images. *J Neurosci Methods* **177**, 497–509 (2009).

[39] Qiu, J. & Li, F.-F. Quantitative morphological analysis of curvilinear network for microscopic

image based on individual fibre segmentation. *J Microsc* **256**, 153–165 (2014).

[40] Baumgarten, W. & Hauser, M. J. Computational algorithms for extraction and analysis of two-dimensional transportation networks. *J Comput Interdiscip Sci* **3**, 107–16 (2012).

[41] Obara, B., Grau, V. & Fricker, M. D. A bioimage informatics approach to automatically extract complex fungal networks. *Bioinformatics* **28**, 2374–2381 (2012).

[42] Garey, M. R., Johnson, D. S. & Tarjan, R. E. The planar Hamiltonian circuit problem is NP-complete. *Siam J Comput* **5**, 704–714 (1976).

[43] Schrijver, A. *Theory of Linear and Integer Programming* (Wiley, New York, 1998), 1 edn.

[44] Linderoth, J. T. & Ralphs, T. K. Noncommercial software for mixed-integer linear programming. *Int Prog Theor Pract* **3**, 253–303 (2005).

[45] Saporta, G. & Youness, G. Comparing two partitions: some proposals and experiments. In *Compstat*, 243–248 (Springer, Berlin, 2002).

[46] Meilă, M. Comparing clusterings: an axiomatic view. In *Proc 22nd Internat Conf Mach Learn*, ICML '05, 577–584 (ACM, New York, 2005).

[47] Denœud, L. & Guénoche, A. Comparison of distance indices between partitions. In *Data Science and Classification*, 21–28 (Springer, Berlin, 2006).

[48] Lancichinetti, A. & Fortunato, S. Community detection algorithms: a comparative analysis. *Phys Rev E* **80**, 056117 (2009).

[49] Breuer, D. *et al.* Quantitative analyses of the plant cytoskeleton reveal underlying organizational principles. *J R Soc Interface* **11**, 20140362 (2014).

[50] Brandner, D. & Withers, G. Development of the axon and dendritic arbors in cultured hippocampal neurons. CC-BY 3.0, http://www.cellimagelibrary.org/contributors/742096, Date of access: 07/09/2015 (2014).

[51] Masland, R. H. The fundamental plan of the retina. *Nat Neurosci* **4**, 877–886 (2001).

[52] Katifori, E. & Magnasco, M. O. Quantifying loopy network architectures. *PLoS One* **7**, e37994 (2012).

[53] Karp, R. M. *Reducibility among combinatorial problems* (Springer, New York, 1972), 1 edn.

[54] Himsolt, M. GML: a portable graph file format. http://www.fmi.uni-passau.de/graphlet/gml/gml-tr.html, Date of access: 07/09/2015 (1997).

[55] Kuhn, H. W. The Hungarian method for the assignment problem. *Nav Res Logist Q* **2**, 83–97 (1955).

[56] Wolsey, L. A. & Nemhauser, G. L. *Integer and Combinatorial Optimization* (Wiley-Interscience, New York, 1999), 1 edn.

[57] Hubert, L. & Arabie, P. Comparing partitions. *J Classif* **2**, 193–218 (1985).

[58] Meilă, M. Comparing clusterings by the variation of information. In *Learning Theory and Kernel Machines*, 173–187 (Springer, Berlin, 2003).

[59] Newman, M. Communities, modules and large-scale structure in networks. *Nat Phys* **8**, 25–31 (2012).

[60] Akaike, H. A new look at the statistical model identification. *IEEE Trans Autom Control* **19**, 716–723 (1974).

[61] Burlacu, S., Janmey, P. & Borejdo, J. Distribution of actin filament lengths measured by fluorescence microscopy. *Am J Physiol-Cell Ph* **262**, C569–C577 (1992).

[62] Ermentrout, G. B. & Edelstein-Keshet, L. Models for the length distributions of actin filaments: II. polymerization and fragmentation by gelsolin acting together. *B Math Biol* **60**, 477–503 (1998).

[63] Waller, F. & Nick, P. Response of actin microfilaments during phytochrome-controlled growth of maize seedlings. *Protoplasma* **200**, 154–162 (1997).

[64] Sampathkumar, A. *et al.* Live cell imaging reveals structural associations between the actin and microtubule cytoskeleton in Arabidopsis. *Plant Cell* **23**, 2302–2313 (2011).

[65] Staiger, C. J. *et al.* Actin filament dynamics are dominated by rapid growth and severing activity in the Arabidopsis cortical array. *J Cell Biol* **184**, 269–280 (2009).

[66] Henty-Ridilla, J. L., Li, J., Blanchoin, L. & Staiger, C. J. Actin dynamics in the cortical array of plant cells. *Curr Opin Plant Biol* **16**, 678–687 (2013).

[67] Gardel, M. L. *et al.* Elastic behavior of cross-linked and bundled actin networks. *Science* **304**, 1301–1305 (2004).

[68] Claessens, M. M., Bathe, M., Frey, E. & Bausch, A. R. Actin-binding proteins sensitively mediate F-actin bundle stiffness. *Nat Mater* **5**, 748–753 (2006).

[69] Sousbie, T., Pichon, C., Colombi, S., Novikov, D. & Pogosyan, D. The 3D skeleton: tracing the filamentary structure of the universe. *Mon Not R Astron Soc* **383**, 1655–1670 (2008).

[70] Sousbie, T., Pichon, C., Courtois, H., Colombi, S. & Novikov, D. The three-dimensional skeleton of the SDSS. *Astrophys J Lett* **672**, L1 (2008).

[71] Faltenbacher, A., Gottlöber, S., Kerscher, M. & Mueller, V. Correlations in the orientations

of galaxy clusters. *Astron Astrophys* **395**, 1–9 (2002).

[72] Aubert, D., Pichon, C. & Colombi, S. The origin and implications of dark matter anisotropic cosmic infall on L haloes. *Mon Not R Astron Soc* **352**, 376–398 (2004).

[73] Milo, R. *et al.* Network motifs: simple building blocks of complex networks. *Int S Techn Pol Inn* **298**, 824–827 (2002).

[74] Shen-Orr, S., Milo, R., Mangan, S. & Alon, U. Network motifs in the transcriptional regulation network of Escherichia coli. *Nat Genet* **31**, 64–68 (2002).

[75] Sporns, O. & Kötter, R. Motifs in brain networks. *PLoS Biol* **2**, e369 (2004).

[76] Paredez, A. R., Somerville, C. R. & Ehrhardt, D. W. Visualization of cellulose synthase demonstrates functional association with microtubules. *Int S Techn Pol Inn* **312**, 1491–1495 (2006).

[77] Riedl, J. *et al.* Lifeact: a versatile marker to visualize F-actin. *Nat Methods* **5**, 605–607 (2008).

[78] Tero, A. *et al.* Rules for biologically inspired adaptive network design. *Int S Techn Pol Inn* **327**, 439–442 (2010).

[79] Barthélemy, M. Spatial networks. *Phys Rep* **499**, 1–101 (2011).

[80] Gittes, F., Mickey, B., Nettleton, J. & Howard, J. Flexural rigidity of microtubules and actin filaments measured from thermal fluctuations in shape. *J Cell Biol* **120**, 923–934 (1993).

[81] van Mameren, J., Vermeulen, K. C., Gittes, F. & Schmidt, C. F. Leveraging single protein polymers to measure flexural rigidity. *J Phys Chem B* **113**, 3837–3844 (2009).

[82] Ali, M. & Brocchini, S. Synthetic approaches to uniform polymers. *Adv Drug Delivery Rev* **58**, 1671–1687 (2006).

[83] Hartmann, L. & Börner, H. Precision polymers: monodisperse, monomer-sequence-defined segments to target future demands of polymers in medicine. *Adv Mater* **21**, 3425–3431 (2009).

[84] Meijering, E. Neuron tracing in perspective. *Cytometry A* **77**, 693–704 (2010).

[85] Inoue, K. Functional dendrimers, hyperbranched and star polymers. *Prog Polym Sci* **25**, 453–571 (2000).

[86] Tomalia, D. A. & Frechet, J. M. *Dendrimers and other dendritic polymers* (Wiley, New York, 2001), 1 edn.

[87] Verwer, R. W. & van Pelt, J. A new method for the topological analysis of neuronal tree structures. *J Neurosci Meth* **8**, 335–351 (1983).

[88] Ascoli, G. A., Donohue, D. E. & Halavi, M. Neuromorpho.org: a central resource for neuronal

morphologies. *J Neurosci* **27**, 9247–9251 (2007).

[89] Tarsi, M. On the decomposition of a graph into stars. *Discrete Math* **36**, 299–304 (1981).

[90] Cohen, E. & Tarsi, M. NP-completeness of graph decomposition problems. *J Complexity* **7**, 200–212 (1991).

[91] Lin, C. & Shyu, T.-W. A necessary and sufficient condition for the star decomposition of complete graphs. *J Graph Theor* **23**, 361–364 (1996).

[92] Even, G., Garg, N., Könemann, J., Ravi, R. & Sinha, A. Min–max tree covers of graphs. *Op Res Lett* **32**, 309–315 (2004).

[93] Horak, P. & McAvaney, K. On covering vertices of a graph by trees. *Discrete Math* **308**, 4414–4418 (2008).

[94] Croton, D. J. Damn you, little h! (or, real-world applications of the hubble constant using observed and simulated data). *Publ Astron Soc Aust* **30**, e052 (2013).

[95] Rao Arikati, S. & Pandu Rangan, C. Linear algorithm for optimal path cover problem on interval graphs. *Inform Process Lett* **35**, 149–153 (1990).

[96] Andreatta, G. & Mason, F. Path covering problems and testing of printed circuits. *Discrete Appl Math* **62**, 5–13 (1995).

[97] Lin, R., Olariu, S. & Pruesse, G. An optimal path cover algorithm for cographs. *Comput Math Appl* **30**, 75–83 (1995).

[98] Pak-Ken, W. Optimal path cover problem on block graphs. *Lect Notes Comput Sc* **225**, 163–169 (1999).

[99] Lin, G., Cai, Z. & Lin, D. Vertex covering by paths on trees with its applications in machine translation. *Inform Process Lett* **97**, 73–81 (2006).

[100] Brešar, B., Kardoš, F., Katrenič, J. & Semanišin, G. Minimum k-path vertex cover. *Discrete Appl Math* **159**, 1189–1195 (2011).

[101] Buchin, K., Knauer, C., Kriegel, K., Schulz, A. & Seidel, R. On the number of cycles in planar graphs. In *Computing and Combinatorics*, 97–107 (Springer, Berlin, 2007).

[102] Biswas, S., Durocher, S., Mondal, D. & Nishat, R. I. Hamiltonian paths and cycles in planar graphs. In *Combinatorial Optimization and Applications*, Lecture Notes in Computer Science, 83–94 (Springer, Berlin, 2012).

[103] Garey, M. R. & Johnson, D. S. *Computers and intractability: a guide to NP-completeness* (WH Freeman, New York, 1979), 1 edn.

[104] Bertossi, A. A. The edge Hamiltonian path problem is NP-complete. *Inform Proc Lett* **13**, 157–159 (1981).

[105] Vazirani, V. V. *Approximation algorithms* (Springer, Berlin, 2001), 1 edn.

[106] Wu, T.-H. A note on a global approach for general 0–1 fractional programming. *Eur J Oper Res* **101**, 220–223 (1997).

[107] Yue, D., Guillén-Gosálbez, G. & You, F. Global optimization of large-scale mixed-integer linear fractional programming problems: a reformulation-linearization method and process scheduling applications. *AIChE J* **59**, 4255–4272 (2013).

[108] Griva, I., Nash, S. G. & Sofer, A. *Linear and nonlinear optimization* (SIAM, Philadelphia, 2009), 2 edn.

[109] Van Rossum, G. & Drake Jr, F. L. *Python Language Reference Manual* (Network Theory Ltd, Godalming, 2011), 1 edn.

[110] Oliphant, T. E. Python for scientific computing. *Comput Sci Engineer* **9**, 10–20 (2007).

[111] Hagberg, A., Swart, P. & S Chult, D. Exploring network structure, dynamics, and function using NetworkX. In *Proc 7th Python Sci Conf* (LANL, Pasadena, 2008).

[112] Dahl, J. & Vandenberghe, L. Cvxopt: a Python package for convex optimization. http://abel.ee.ucla.edu/cvxopt/, Date of access: 07/09/2015 (2006).

[113] Finlay, J. Pygtk 2.0 tutorial. http://www.pygtk.org/dist/pygtk2-tut.pdf, Date of access: 07/09/2015 (2005).

[114] Frangi, A. F., Niessen, W. J., Vincken, K. L. & Viergever, M. A. Multiscale vessel enhancement filtering. In *Med Image Comput Comput-Ass Interv*, Lecture Notes in Computer Science, 130–137 (Springer, Berlin, 1998).

[115] Haralick, R. M., Sternberg, S. R. & Zhuang, X. Image analysis using mathematical morphology. *IEEE Trans Pattern Anal Mach Intell* 532–550 (1987).

[116] Toussaint, G. T. The relative neighbourhood graph of a finite planar set. *Lect Notes Comput Sci* **12**, 261–268 (1980).

[117] Supowit, K. J. The relative neighborhood graph, with an application to minimum spanning trees. *J ACM* **30**, 428–448 (1983).

[118] Goldberg, M. K., Hayvanovych, M. & Magdon-Ismail, M. Measuring similarity between sets of overlapping clusters. In *IEEE 2nd Internat Conf Social Comput*, 303–308 (IEEE, Minneapolis, 2010).

[119] Lancichinetti, A., Fortunato, S. & Kertész, J. Detecting the overlapping and hierarchical community structure in complex networks. *New J Phys* **11**, 033015 (2009).

[120] Lancichinetti, A. & Fortunato, S. Benchmarks for testing community detection algorithms on directed and weighted graphs with overlapping communities. *Phys Rev E* **80**, 016118 (2009).

[121] Zhou, D., Li, J. & Zha, H. A new mallows distance based metric for comparing clusterings. In *Proc 22nd Internat Conf Mach Learn*, 1028–1035 (ACM, New York, 2005).

[122] Bae, E., Bailey, J. & Dong, G. A clustering comparison measure using density profiles and its application to the discovery of alternate clusterings. *Data Min Knowl Disc* **21**, 427–471 (2010).

[123] Coen, M. H., Ansari, M. H. & Fillmore, N. Comparing clusterings in space. In *Proc 27th Internat Conf Mach Learn*, 231–238 (Omnipress, Madison, 2010).

[124] Kasza, K. E. *et al.* Actin filament length tunes elasticity of flexibly cross-linked actin networks. *Biophys J* **99**, 1091–1100 (2010).

[125] Bai, M., Missel, A. R., Levine, A. J. & Klug, W. S. On the role of the filament length distribution in the mechanics of semiflexible networks. *Acta Biomater* **7**, 2109–2118 (2011).
